# Supplementary material for: Establishing a standard surgery for esophagogastric junction cancer: Final results from the JGCA-JES nationwide prospective study
Source: Cell Rep Med. 2026 Feb 17;7(2):102627. doi: 10.1016/j.xcrm.2026.102627 (PMC12923963; doi:10.1016/j.xcrm.2026.102627)
Supplement: Document S1. Figures S1–S3 and Tables S1–S4 [file mmc1.pdf]

Cell Reports Medicine, Volume *7*

Supplemental information

Establishing a standard surgery for esophagogastric junction cancer: Final results from the JGCA-JES nationwide prospective study

Yukinori Kurokawa, Hiroya Takeuchi, Yuichiro Doki, Shinji Mine, Masanori Terashima, Takushi Yasuda, Kazuhiro Yoshida, Hiroyuki Daiko, Shinichi Sakuramoto, Takaki Yoshikawa, Chikara Kunisaki, Yasuyuki Seto, Shigeyuki Tamura, Toshio Shimokawa, Takeshi Sano, and Yuko Kitagawa

Supplemental information

Establishing a standard surgery for esophagogastric junction cancer: Final results from the JGCA-JES nationwide prospective study

Yukinori Kurokawa, Hiroya Takeuchi, Yuichiro Doki, Shinji Mine, Masanori Terashima, Takushi Yasuda, Kazuhiro Yoshida, Hiroyuki Daiko, Shinichi Sakuramoto, Takaki Yoshikawa, Chikara Kunisaki, Yasuyuki Seto, Shigeyuki Tamura, Toshio Shimokawa, Takeshi Sano, Yuko Kitagawa

Table S1. Site of first recurrence in 142 patients with recurrence

Sites	n (%)
Lymph nodes	63 (44.4 %)
Abdominal paraaortic	39 (27.5 %)
Mediastinal	22 (15.5 %)
Cervical	11 (7.7 %)
Perigastric	7 (4.9 %)
Others	5 (3.5 %)
Liver	37 (26.1 %)
Peritoneum	31 (21.8 %)
Lung	30 (21.1 %)
Pleura	11 (7.7 %)
Bone	9 (6.3 %)
Anastomotic site	7 (4.9 %)
Adrenal gland	5 (3.5 %)
Brain	4 (2.8 %)
Remnant esophagus	3 (2.1 %)
Others	4 (2.8 %)

Some cases are counted in multiple sites.

Table S2. Metastasis rates and therapeutic efficacy indices in the mediastinal and abdominal nodes the abdominal nodes

	Metastasis Rate (%)	5-year OS in metastatic cases (%)	TEI for OS	5-year RFS in metastatic cases (%)	TEI for RFS
Upper mediastinal nodes [a]	(n = 117)				
105	0.9	0.0	0	0.0	0
106L	2.6	66.7	1.7	33.3	0.9
106R	5.1	33.3	1.7	16.7	0.9
Middle mediastinal nodes [a]	(n = 117)				
107	2.6	0.0	0	0.0	0
108	6.8	50.0	**3.4**	25.0	1.7
109L	3.4	25.0	0.9	25.0	0.9
109R	3.4	25.0	0.9	25.0	0.9
Lower mediastinal nodes	(n = 347)				
110	9.2	37.5	**3.5**	15.6	1.4
111	3.5	41.7	1.4	25.0	0.9
112	2.0	28.6	0.6	14.3	0.3
Proximal perigastric nodes	(n = 350)				
1	34.9	48.4	**16.9**	36.9	**12.9**
2	27.1	38.9	**10.6**	28.4	**7.7**
3	37.1	50.8	**18.9**	33.8	**12.6**
7	23.7	37.3	**8.9**	24.1	**5.7**
Left greater curvature nodes [b]	(n = 174)				
4sa	4.6	25.0	1.1	12.5	0.6
4sb	1.1	0.0	0	0.0	0
Distal perigastric nodes [b]	(n = 174)				
4d	1.7	0.0	0	0.0	0
5	1.1	0.0	0	0.0	0
6	1.7	33.3	0.6	0.0	0
Suprapancreatic nodes	(n = 350)				
8a	7.2	40.0	2.9	32.0	**2.3**
9	12.3	30.2	**3.7**	25.6	**3.1**
11p	13.8	37.5	**5.2**	29.2	**4.0**
11d	4.3	26.7	1.2	6.7	0.3
Abdominal hiatal nodes	(n = 346)				
19	5.2	22.2	1.2	11.1	0.6
20	4.0	35.7	1.4	21.4	0.9

Paraaortic nodes	(n = 340)				
16a2	4.4	13.3	0.6	0.0	0

TEI, therapeutic efficacy index; OS, overall survival; RFS, recurrence-free survival.

Indices in bold indicate values greater than 3 for 5-year OS and 2 for 5-year RFS.

[a] Values in the upper or middle mediastinal nodes were evaluated in 117 patients who underwent subtotal esophagectomy via right transthoracic approach.

[b] Values in the left greater curvature or distal perigastric nodes were evaluated in 174 patients who underwent total gastrectomy.

1 indicates right para-cardial node; 2, left para-cardial node; 3, lesser curvature node; 4sa, left greater curvature node along the short gastric arteries; 4sb, left greater curvature node along the left gastroepiploic artery; 4d, right greater curvature node; 5, supra-pyloric node; 6, infra-pyloric node; 7, node along the trunk of left gastric artery; 8a, antero-superior node along the common hepatic artery; 9, celiac artery node; 11p, proximal splenic artery node; 11d, distal splenic artery node; 19, infra-diaphragmatic node predominantly along the subphrenic artery; 20, para-esophageal node in the diaphragmatic esophageal hiatus; 16a2, para-aortic node between the upper margin of the origin of the celiac artery and the lower border of the left renal vein; 105, upper thoracic para-esophageal node; 106L, left recurrent laryngeal nerve node; 106R, right recurrent laryngeal nerve node; 107, subcarinal node; 108, middle thoracic para-esophageal node; 109L, left main bronchus node; 109R, right main bronchus node; 110, lower thoracic para-esophageal node; 111, supra-diaphragmatic node; 112, posterior mediastinal node.

Table S3. Therapeutic efficacy indices for 5-year overall survival and recurrence-free survival in the abdominal nodes according to the length of gastric involvement

Lymph node station	Gastric involvement [b]		
	≤2.0 cm	2.1–4.0 cm	>4.0 cm
	OS / RFS	OS / RFS	OS / RFS
Proximal perigastric nodes	(n = 145)	(n = 141)	(n = 64)
1	**16.6** / **11.0**	**14.9** / **12.1**	**21.9** / **18.8**
2	**11.0** / **7.6**	**11.3** / **8.5**	**7.8** / **6.3**
3	**17.2** / **10.3**	**19.9** / **13.5**	**20.3** / **15.6**
7	**8.3** / **5.5**	**9.2** / **5.7**	**9.4** / **6.3**
Left greater curvature nodes [a]	(n = 44)	(n = 78)	(n = 52)
4sa	0 / 0	1.3 / 1.3	1.9 / 0
4sb	0 / 0	0 / 0	0 / 0
Distal perigastric nodes [a]	(n = 44)	(n = 78)	(n = 52)
4d	0 / 0	0 / 0	0 / 0
5	0 / 0	0 / 0	0 / 0
6	0 / 0	1.3 / 0	0 / 0
Suprapancreatic nodes	(n = 145)	(n = 141)	(n = 64)
8a	2.8 / **2.8**	**3.6** / **2.1**	1.6 / 1.6
9	2.8 / 1.4	**5.7** / **5.7**	1.6 / 1.6
11p	2.1 / **2.1**	**5.7** / **4.3**	**10.9** / **7.8**
11d	0 / 0	0.7 / 0	**4.7** / 1.6
Abdominal hiatal nodes	(n = 145)	(n = 138)	(n = 63)
19	2.1 / 0.7	0 / 0	1.6 / 1.6
20	0.7 / 0	2.9 / **2.2**	0 / 0
Paraaortic nodes	(n = 141)	(n = 137)	(n = 62)
16a2	0.7 / 0	0 / 0	1.6 / 0

OS, overall survival; RFS, recurrence-free survival.

Indices in bold indicate values greater than 3 for 5-year OS and greater than 2 for 5-year RFS.

[a] Indices in the left greater curvature or distal perigastric nodes were evaluated in 174 patients who underwent total gastrectomy.

[b] The length of gastric involvement was measured in the resected specimen.

1 indicates right para-cardial node; 2, left para-cardial node; 3, lesser curvature node; 4sa, left greater curvature node along the short gastric arteries; 4sb, left greater curvature node along the left gastroepiploic artery; 4d, right greater curvature node; 5, supra-pyloric node; 6, infra-pyloric node; 7, node along the trunk of left gastric artery; 8a, antero-superior node along the common hepatic artery; 9, celiac artery node; 11p, proximal splenic artery node; 11d, distal splenic artery node; 19, infra-diaphragmatic node predominantly along the subphrenic artery; 20, para-esophageal node in the diaphragmatic esophageal hiatus; 16a2, para-aortic node between the upper margin of the origin of the celiac artery and the lower border of the left renal vein.

Table S4. Therapeutic efficacy indices for 5-year overall survival and recurrence-free survival in patients with cN+ in the upper or middle mediastinal field

	OS / RFS
Upper mediastinal nodes	(n = 30)
105	0 / 0
106L	**6.7** / **3.3**
106R	**3.3** / 0
Middle mediastinal nodes	(n = 30)
107	0 / 0
108	**10.0** / **6.7**
109L	0 / 0
109R	**3.3** / **3.3**

OS, overall survival; RFS, recurrence-free survival.

Indices in bold indicate values greater than 3 for 5-year OS and greater than 2 for 5-year RFS.

105 indicates upper thoracic para-oesophageal node; 106L, left recurrent laryngeal nerve node; 106R, right recurrent laryngeal nerve node; 107, subcarinal node; 108, middle thoracic para-oesophageal node; 109L, left main bronchus node; 109R, right main bronchus node.

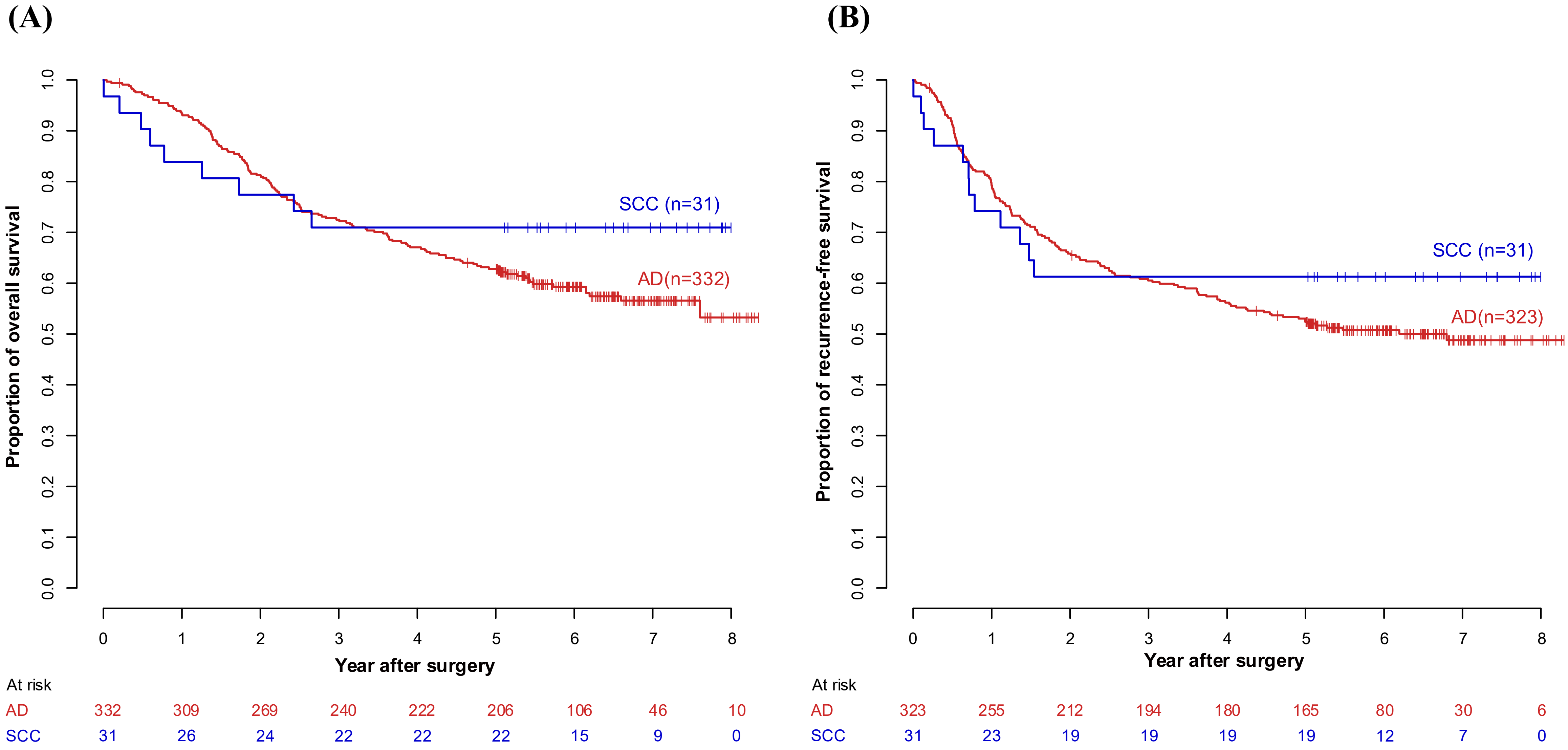

Figure S1. Overall survival in all eligible patients (A) and recurrence-free survival in all eligible patients who achieved R0 or R1 resection (B) by histological types. AD, adenocarcinoma; SCC, squamous cell carcinoma.

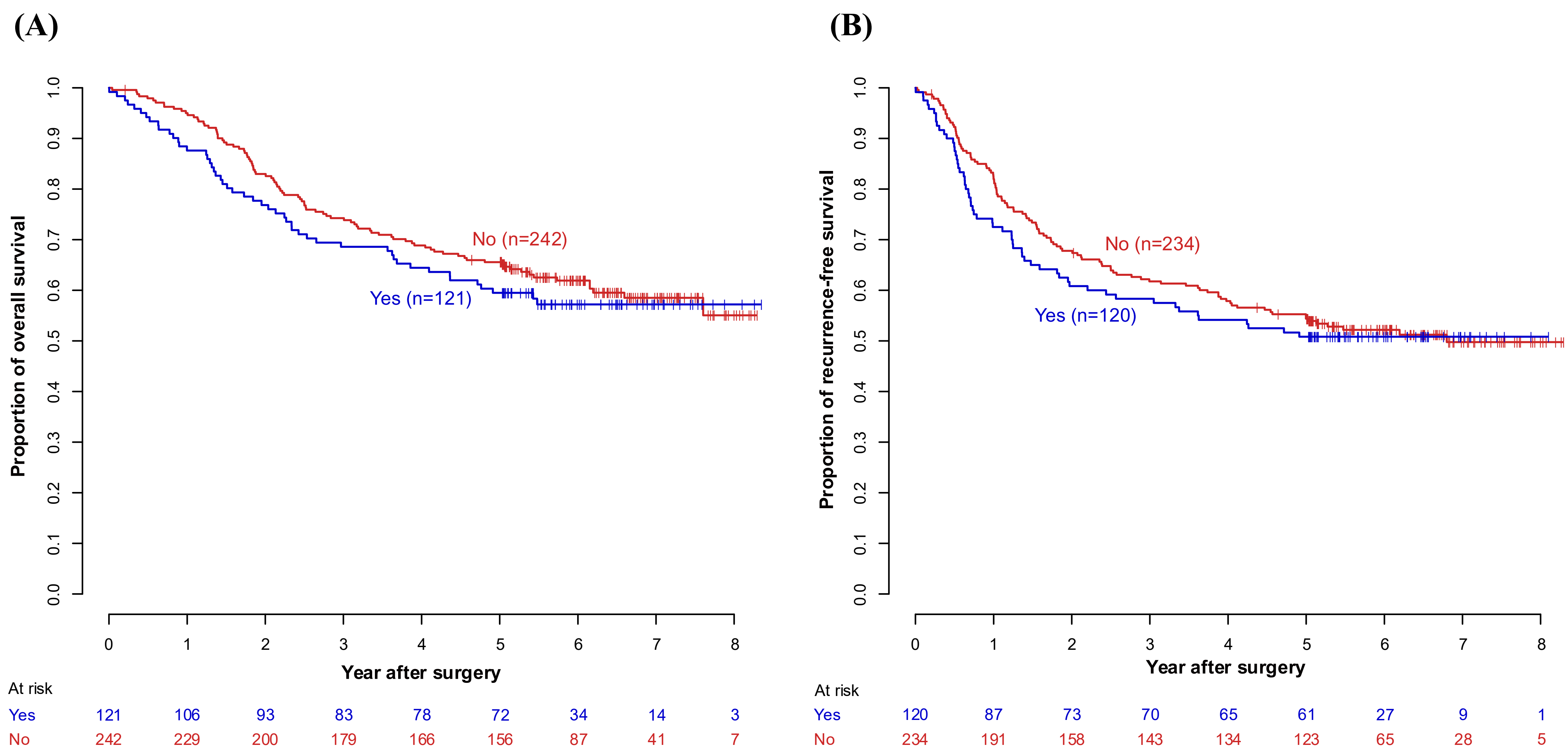

Figure S2. Overall survival in all eligible patients (A) and recurrence-free survival in all eligible patients who achieved R0 or R1 resection (B) by the presence or absence of neoadjuvant chemotherapy.

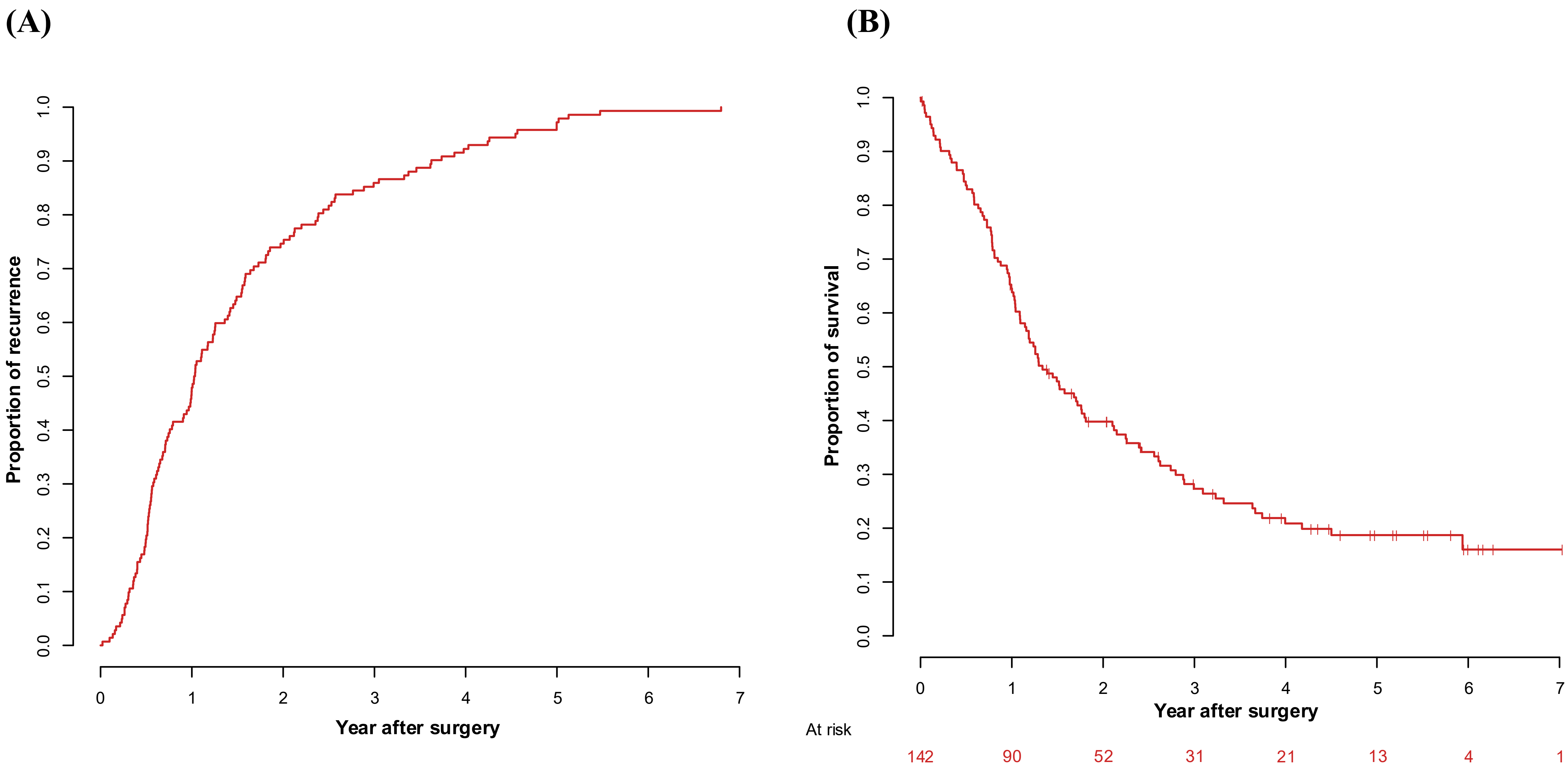

Figure S3. Cumulative recurrence rate (A) and proportion of survival after recurrence (B) in patients who experienced tumor recurrence.

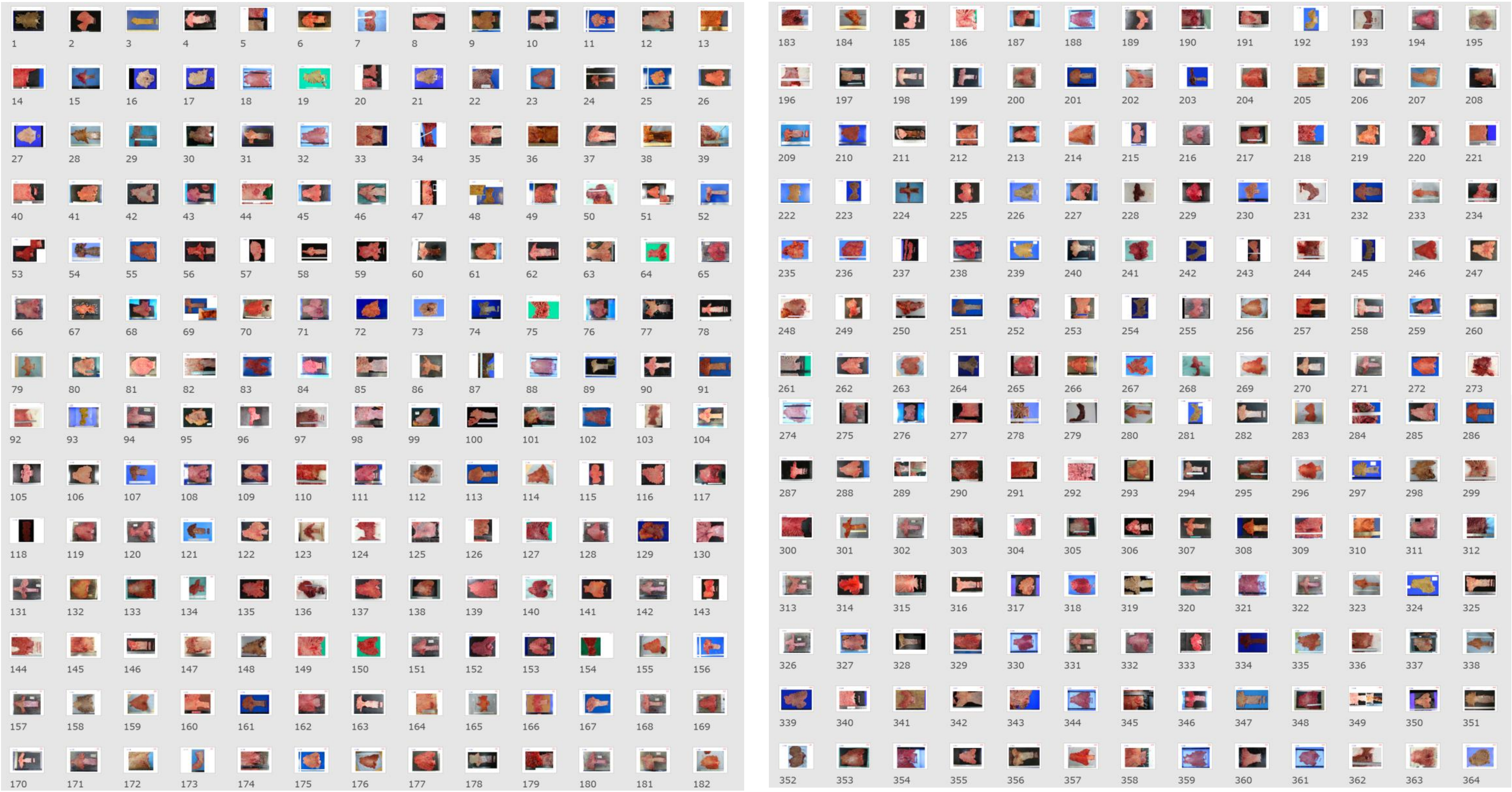

Figure S4. Representative photographs of resected specimens from each case, collected for central eligibility review.
