# Supplementary material for: Establishing a standard surgery for esophagogastric junction cancer: Final results from the JGCA-JES nationwide prospective study
Source: Cell Rep Med. 2026 Feb 17;7(2):102627. doi: 10.1016/j.xcrm.2026.102627 (PMC12923963; doi:10.1016/j.xcrm.2026.102627)
Supplement: Document S2. Article plus supplemental information [file mmc2.pdf]

Cell Reports
Medicine

Article

Establishing a standard surgery for esophagogastric junction cancer: Final results from the JGCA-JES nationwide prospective study

Graphical abstract

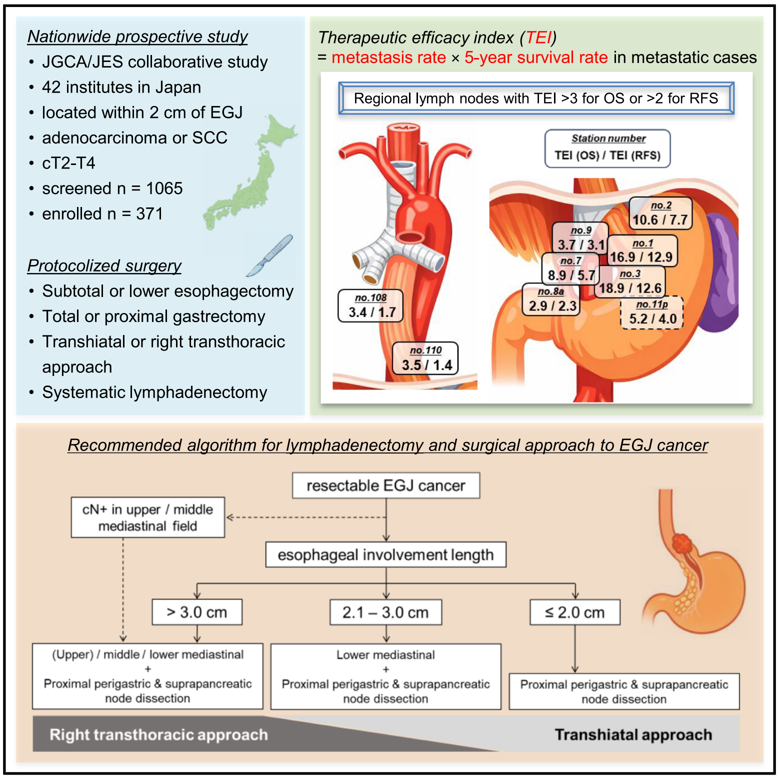

Highlights

- Abdominal station nos. 1, 2, 3, 7, 9, and 11p show high TEIs
- TEIs in mediastinal station nos. 108 and 110 increase with esophageal involvement
- All other nodes, including distal perigastric and paraaortic nodes, show low TEIs
- Lymphadenectomy should be tailored to esophageal involvement length in EGJ cancer

Authors

Yukinori Kurokawa, Hiroya Takeuchi, Yuichiro Doki, ..., Toshio Shimokawa, Takeshi Sano, Yuko Kitagawa

Correspondence

ykurokawa@gesurg.med.osaka-u.ac.jp

In brief

Kurokawa et al. examine the therapeutic efficacy index (TEI) across all regional lymph nodes in esophagogastric junction cancer using a nationwide multicenter prospective study. Proximal perigastric and suprapancreatic nodes show a high TEI, whereas TEIs in middle and lower para-esophageal stations vary according to the length of esophageal involvement.

Kurokawa et al., 2026, Cell Reports Medicine 7, 102627
February 17, 2026 © 2026 The Authors. Published by Elsevier Inc.
https://doi.org/10.1016/j.xcrm.2026.102627

CellPress

Cell Reports Medicine

CellPress
OPEN ACCESS

Article

Establishing a standard surgery for esophagogastric junction cancer: Final results from the JGCA-JES nationwide prospective study

Yukinori Kurokawa,[1,15,*] Hiroya Takeuchi,[2,3] Yuichiro Doki,[1] Shinji Mine,[4] Masanori Terashima,[5] Takushi Yasuda,[6] Kazuhiro Yoshida,[7] Hiroyuki Daiko,[8] Shinichi Sakuramoto,[9] Takaki Yoshikawa,[10] Chikara Kunisaki,[11] Yasuyuki Seto,[12] Shigeyuki Tamura,[13] Toshio Shimokawa,[14] Takeshi Sano,[4] and Yuko Kitagawa[3]

[1]Department of Gastroenterological Surgery, The University of Osaka Graduate School of Medicine, Osaka, Japan
[2]Department of Surgery, Hamamatsu University School of Medicine, Hamamatsu, Japan
[3]Department of Surgery, Keio University School of Medicine, Tokyo, Japan
[4]Department of Surgery, Cancer Institute Hospital, Tokyo, Japan
[5]Department of Gastric Surgery, Shizuoka Cancer Center, Mishima, Japan
[6]Department of Surgery, Kindai University Faculty of Medicine, Osaka, Japan
[7]Department of Gastroenterological and Pediatric Surgery, Gifu University School of Medicine, Gifu, Japan
[8]Department of Esophageal Surgery, National Cancer Center Hospital East, Kashiwa, Japan
[9]Department of Gastroenterological Surgery, Saitama Medical University International Medical Center, Saitama, Japan
[10]Department of Surgery, Kanagawa Cancer Center, Yokohama, Japan
[11]Department of Surgery, Yokohama City University Gastroenterological Center, Yokohama, Japan
[12]Department of Gastrointestinal Surgery, The University of Tokyo, Tokyo, Japan
[13]Department of Surgery, Kansai Rosai Hospital, Amagasaki, Japan
[14]Department of Biostatistics, Faculty of Medicine Wakayama Medical University, Wakayama, Japan
[15]Lead contact
*Correspondence: ykurokawa@gesurg.med.osaka-u.ac.jp
https://doi.org/10.1016/j.xcrm.2026.102627

SUMMARY

The optimal surgical approach and extent of lymph node dissection for esophagogastric junction (EGJ) cancer remains uncertain. We conduct a nationwide multicenter prospective study in patients with resectable cT2–T4 adenocarcinoma or squamous cell carcinoma with the tumor epicenter located within 2 cm of the EGJ. Patients undergo subtotal or lower esophagectomy with dissection of all regional lymph nodes. Of 1,065 patients screened, 371 are enrolled before surgery. Final analysis shows that proximal perigastric and suprapancreatic nodes exhibit a high therapeutic efficacy index (TEI), strongly supporting their dissection for improved long-term survival. TEIs in middle and lower para-esophageal stations are higher when esophageal involvement exceeds 3 and 2 cm, respectively. Conversely, all other stations, including distal perigastric and paraaortic nodes, have low TEIs, indicating minimal survival impact. Thus, mediastinal node dissection should be tailored to esophageal involvement length. This study is registered at UMIN Clinical Trials Registry (UMIN000013205).

INTRODUCTION

Esophagogastric junction (EGJ) cancer has long been a serious malignancy in Western countries, and in recent years, its incidence has been increasing rapidly in Eastern countries as well.[1–3] Due to the tumor's location at the transition between the thoracic and abdominal cavities, its metastatic pattern is highly complex, and no universally accepted standard surgical approach has been established for this disease.[4–6] Traditionally, surgeons have determined the extent of lymph node dissection in cancer surgery based on the metastatic rates of individual lymph nodes. However, a critical limitation of this approach is that most studies evaluating metastatic rates are retrospective in nature, introducing selection bias—lymph nodes more likely to harbor metastases tend to be dissected, while those deemed less likely are often omitted.

Conducting randomized controlled trials (RCTs) is the most reliable way to minimize selection bias. In the 1990s, two RCTs were conducted in patients with EGJ cancer to compare different surgical approaches. One was a Dutch trial that compared transhiatal (TH) subtotal esophagectomy with right transthoracic (RT) subtotal esophagectomy for Siewert type I or II EGJ adenocarcinoma.[7,8] However, the final results were inconclusive, and no standard surgical approach was established. The other was a Japanese trial (JCOG9502) comparing left thoracoabdominal lower esophagectomy and abdominal TH lower esophagectomy for gastric or Siewert type II/III EGJ adenocarcinoma,[9,10] which concluded that lower esophagectomy via the abdominal TH

© 2026 The Authors. Published by Elsevier Inc.
This is an open access article under the CC BY license (http://creativecommons.org/licenses/by/4.0/).

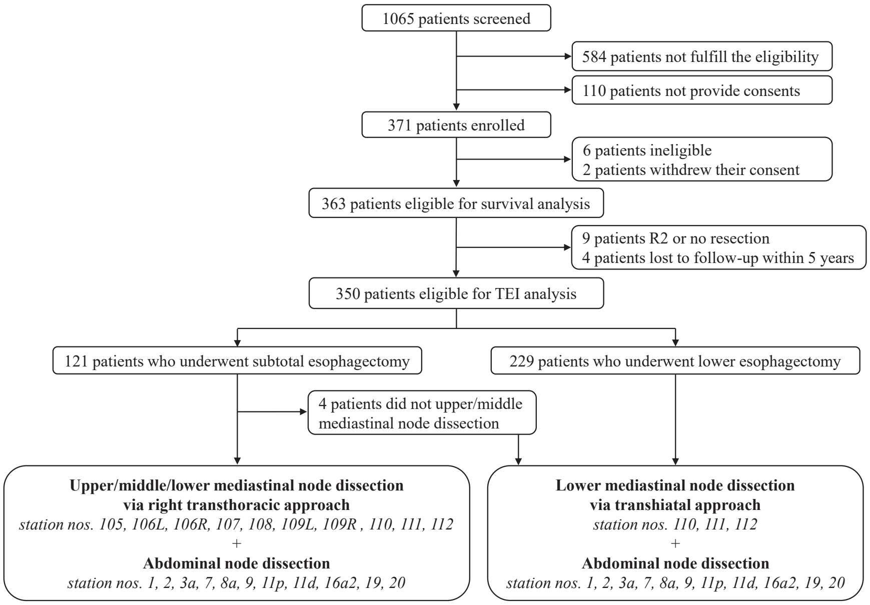

Figure 1. Flow diagram of patients in this study

approach is the standard for adenocarcinomas with ≤3 cm of esophageal involvement. Unfortunately, this trial excluded patients with esophageal involvement exceeding 3 cm, who constitute a representative subset of EGJ adenocarcinoma patients. Furthermore, these earlier trials compared only two surgical approaches without evaluating the optimal extent of lymph node dissection within each. In addition, patients with another histological type—squamous cell carcinoma (SCC)—were not included in these trials, although recent landmark trials on perioperative treatment for EGJ cancer have included both histological types together.[11–13] As a result, the optimal extent of lymphadenectomy for EGJ cancer remains undefined.

To resolve this challenge, the Japanese Gastric Cancer Association (JGCA) and the Japan Esophageal Society (JES) jointly launched a nationwide multicenter prospective study in 2014 to establish a standardized surgery for EGJ cancer.[14–16] The initial analysis of this study provided accurate metastatic rates for both mediastinal and abdominal lymph nodes. On the basis of these findings, JGCA and JES provisionally recommended the extent of lymph node dissection and the surgical approach in their respective gastric and esophageal cancer guidelines.[17,18] However, a fundamental limitation of this provisional algorithm is that, while it identifies lymph nodes with high metastatic rates, it does not determine whether their dissection improves patient outcomes. In other words, even if a metastatic lymph node is removed, dissection may be futile if the disease has already disseminated to other organs.

To further clarify the clinical impact of lymph node dissection, our prospective study predefined a key secondary endpoint: the therapeutic efficacy index (TEI), which is calculated by multiplying the metastasis rate of each lymph node by the 5-year survival rate in metastatic cases.[19] This index reflects the expected improvement in the 5-year survival rate attributable to lymph node dissection. The TEI has been previously used to determine the optimal extent of lymph node dissection in gastric cancer surgery,[20] and it has also been used to examine the extent of dissection for various types of cancers in recent years.[21–25] In this report, we present the long-term outcomes from the final analysis, with a minimum follow-up of 5 years, along with the TEI. Based on these findings, we update the recommended algorithm for lymph node dissection and surgical approach in EGJ cancer.

RESULTS

Patients

Of the 1,065 patients with EGJ cancer screened for this study, 584 did not meet the eligibility criteria and 110 declined to provide consent for enrollment (Figure 1). Ultimately, 371 patients were enrolled between April 22, 2014, and September 29, 2017. After enrollment, two patients withdrew consent, and six patients were deemed ineligible due to non-EGJ cancers (n = 4), a prior history of gastrectomy (n = 1), or inadequate organ function (low platelet count, n = 1). The remaining 363 patients were eligible for this study. The baseline characteristics of these patients are shown in Table 1. More than 80% had Siewert type II adenocarcinoma, while the proportions of Siewert type I adenocarcinoma and SCC were both 8.5%. One-third of all patients received neoadjuvant chemotherapy before surgery. Approximately two-thirds of patients underwent lower esophagectomy via the abdominal TH approach, while one-third underwent subtotal esophagectomy via the RT approach. The proportions of patients who underwent total gastrectomy and proximal or upper gastrectomy were approximately equal. The median number of dissected lymph nodes on pathological examination was 48 overall (46 in the TH approach and 53.5 in the RT approach). R0 resection was achieved in 93.4% of all patients: 92.8% among those with adenocarcinoma, 100% among those with SCC, 92.9% in the TH approach, and 97.7% in the RT approach. More than half of the patients (53.4%) received adjuvant chemotherapy following R0 or R1 resection.

Long-term survival results

At a median follow-up duration of 6.1 years (interquartile range, 5.4–7.0) for all censored patients, the 5-year overall survival (OS) for all 363 eligible patients was 63.5% (95% confidence interval [CI], 58.3–68.3) (Figure 2A). Of the 339 patients who underwent R0 resection and 15 who underwent R1 resection, 142 experienced tumor recurrence (129 after R0 and 13 after R1), with a 5-year recurrence-free survival (RFS) of 53.2% (95% CI, 47.8–58.2) (Figure 2B). By pathological stage (pStage), 5-year OS and 5-year RFS were 87.3% (95% CI, 77.8–93.0) and 79.6% (95% CI, 68.9–87.0) in pStage I, 79.2% (95% CI,

CellPress
OPEN ACCESS

Table 1. Patient characteristics

		n = 363
Age (years)	Median (IQR)	66 (58–71)
Gender	Male	293 (80.7%)
	Female	70 (19.3%)
Histology	Adenocarcinoma	332 (91.5%)
	Squamous cell carcinoma	31 (8.5%)
Siewert type	I	31 (8.5%)
	II	301 (82.9%)
	N/A (squamous cell carcinoma)	31 (8.5%)
Tumor size (cm)	Median (IQR)	4.6 (3.5–6.0)
Length of esophageal involvement[a] (cm)	Median (IQR)	2.0 (1.0–3.0)
Length of gastric involvement[a] (cm)	Median (IQR)	2.5 (1.5–3.7)
Clinical T status	T2	86 (23.7%)
	T3	161 (44.4%)
	T4	116 (32.0%)
Clinical N status	N0	134 (36.9%)
	N1	131 (36.1%)
	N2	81 (22.3%)
	N3	17 (4.7%)
Neoadjuvant chemotherapy	No	242 (66.7%)
	Yes	121 (33.3%)
Surgical approach	Transhiatal	226 (62.3%)
	Right transthoracic	129 (35.5%)
	Left transthoracic	3 (0.8%)
	Simple laparotomy	5 (1.4%)
Type of esophagectomy	Subtotal	121 (33.3%)
	Lower	236 (65.0%)
	N/A (no resection)	6 (1.7%)
Type of gastrectomy	Proximal or upper	180 (49.6%)
	Total	178 (49.0%)
	N/A (no resection)	5 (1.4%)
Number of dissected lymph nodes[b]	Median (IQR)	48 (37–60)
Pathological T status	T0/TX	16 (4.4%)
	T1	48 (13.2%)
	T2	60 (16.5%)
	T3	175 (48.2%)
	T4	59 (16.3%)
	N/A (no resection)	5 (1.4%)
Pathological N status	N0	111 (30.6%)
	N1	91 (25.1%)
	N2	76 (20.9%)
	N3	80 (22.0%)
	N/A (no resection)	5 (1.4%)
Pathological TNM stage	I (including ypT0 N0)	79 (21.8%)
	II	125 (34.4%)
	III	125 (34.4%)
	IV	34 (9.4%)
Residual tumor	R0	339 (93.4%)
	R1	15 (4.1%)
	R2	9 (2.5%)
Adjuvant treatment	No	160 (44.1%)
	Yes	194 (53.4%)
	N/A (R2 resection)	9 (2.5%)

IQR, interquartile range; N/A, not applicable.
T/N/M status and stage were according to the Japanese Classification of Gastric Carcinoma, 14th edition.
[a]Measured in the resected specimen.
[b]Among 358 patients who underwent surgical resection.

71.0–85.3) and 67.2% (95% CI, 58.2–74.7) in pStage II, and 46.4% (95% CI, 37.5–54.8) and 33.0% (95% CI, 24.9–41.3) in pStage III, respectively. There were no significant differences in long-term survival between adenocarcinoma and SCC (log rank test: $p = 0.274$ for OS; $p = 0.442$ for RFS) (Figure S1). Similarly, when stratified by the presence or absence of neoadjuvant chemotherapy, prognosis did not differ significantly (log rank test: $p = 0.405$ for OS; $p = 0.536$ for RFS) (Figure S2).

Recurrence patterns

Among the 142 patients with recurrence after R0 or R1 resection, the most common site of first recurrence was lymph nodes (44.4%), followed by the liver (26.1%), peritoneum (21.8%), and lung (21.1%) (Table S1). The most frequently affected lymph node region was the nondissected areas of the abdominal para-aortic (27.5%) followed by the mediastinal area (15.5%). Among the patients with lymph node recurrence in the abdominal para-aortic ($n = 39$) or mediastinal area ($n = 22$), 25 (64.1%) and 17 (77.3%), respectively, also developed recurrence at other sites. Approximately three-quarters (74.6%) of recurrences occurred within 2 years after surgery (Figure S3A). Among the 142 patients who experienced recurrence, the median survival time after recurrence was 16.0 months (95% CI, 11.5–20.6) and the proportion of survival beyond 5 years after recurrence was 18.7% (95% CI, 12.3–26.2) (Figure S3B).

TEI

Of the 354 patients who underwent R0 or R1 resection, only four patients were lost to follow-up within 5 years after surgery without confirmation of recurrence; therefore, we calculated the TEI for 5-year OS and RFS in mediastinal and abdominal lymph nodes among the remaining 350 patients (Table S2; Figure 3). Indices for left greater curvature and distal perigastric nodes were assessed in 174 of these patients who underwent total gastrectomy, and for upper and middle mediastinal nodes in 117 patients who underwent subtotal esophagectomy via the RT approach. Station nos. 1, 2, 3, 7, 9, and 11p showed a TEI

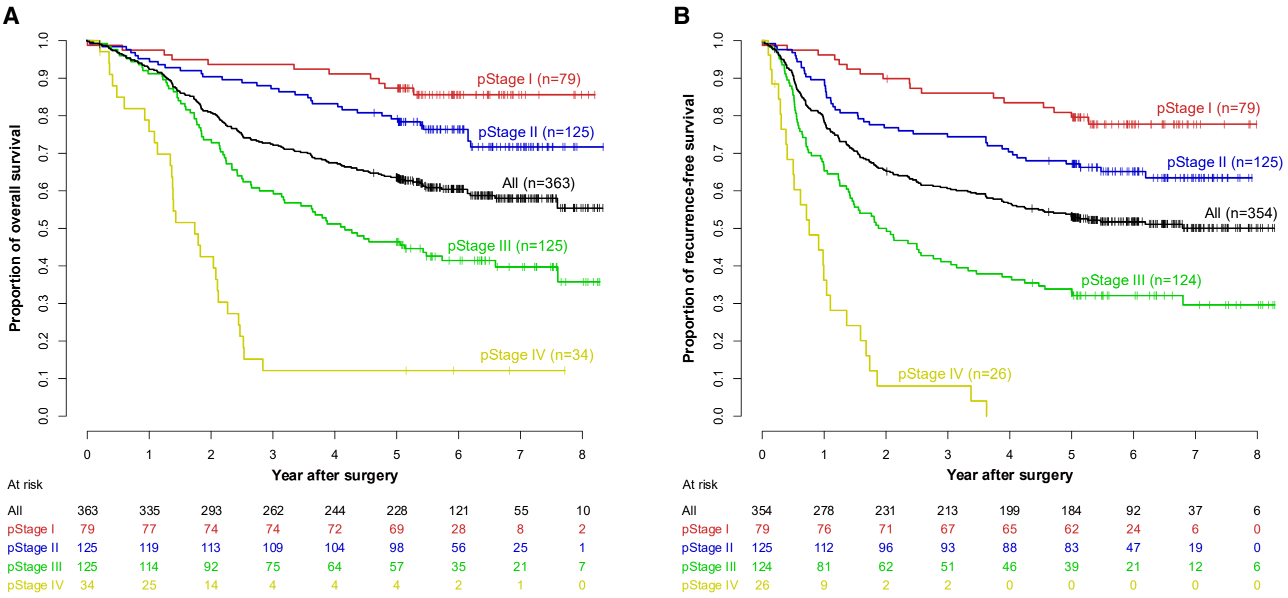

Figure 2. Kaplan–Meier overall and recurrence-free survivals
Overall survival (A) in all eligible patients and recurrence-free survival (B) in all eligible patients who achieved R0 or R1 resection by pathological TNM stages according to the Japanese Classification of Gastric Carcinoma, 14th edition.

exceeding both 3 for 5-year OS and 2 for 5-year RFS, strongly supporting the view that dissection of these nodes can improve long-term survival. Station nos. 8a, 108, and 110 showed a TEI exceeding either 3 for 5-year OS or 2 for 5-year RFS, suggesting that their dissection may improve long-term survival. In contrast, all other nodes had a TEI below both thresholds, indicating minimal impact on long-term survival. The above results were generally consistent even when the analysis was limited to R0 resection cases.

To identify subgroups with a survival benefit—particularly for station nos. 8a, 108, and 110, which showed borderline TEI in the overall cohort—we evaluated TEI differences stratified by the length of esophageal or gastric involvement. In the subgroup with esophageal involvement exceeding 3 cm, station no. 108 demonstrated a high TEI (6.7) for 5-year OS, whereas the subgroup with esophageal involvement $\leq$3 cm showed low TEI in all upper and middle mediastinal nodes (Table 2). For lower mediastinal nodes, the TEI for 5-year OS in station no. 110 increased to 4.9 when esophageal involvement exceeded 2 cm. The TEIs in abdominal nodes were generally unaffected by the length of gastric involvement (Table S3). We also calculated the TEI for 5-year OS and RFS by histological type, but histology did not meaningfully affect TEI at any lymph node station (Table 3). Regarding TEI differences stratified by neoadjuvant or adjuvant chemotherapy, neoadjuvant chemotherapy showed a clear trend toward reducing TEI, while adjuvant chemotherapy increased TEI at most nodes (Table 3). Of particular note, the TEI for 5-year OS at station no. 106L was as high as 5.1 in patients who did not receive neoadjuvant chemotherapy, whereas it dropped to 0 in those who did.

Additionally, we confirmed the efficacy of therapeutic dissection of upper or middle mediastinal nodes in patients diagnosed with cN+ in these regions, since the study protocol mandated thorough mediastinal node dissection when clinical nodal involvement was suspected in this field, regardless of the histological type or the length of esophageal involvement. A total of 30 patients showed cN+ in the upper or middle mediastinal field before initial treatment, and 24 of these patients received neoadjuvant chemotherapy. As a result, several mediastinal nodes, particularly stations 106L and 108, demonstrated a high TEI, suggesting promising efficacy of therapeutic dissection in cN+ cases (Table S4).

Based on these TEIs, we updated the recommended algorithm for the extent of lymph node dissection and the surgical approach for cT2–T4 EGJ cancer (Figure 4).

DISCUSSION

The final analysis of this prospective study successfully elucidated the long-term outcomes of EGJ cancer following surgical resection. Even after R0 resection, approximately 40% of the patients experienced tumor recurrence, primarily in distant lymph nodes, the liver, peritoneum, or lungs. Notably, lymph node recurrence frequently occurred in nondissected areas of the paraaortic or mediastinal regions, often manifesting in multiple locations simultaneously. Based on the TEI analysis, which evaluates the potential for long-term survival following lymph node dissection, routine dissection of proximal perigastric and suprapancreatic lymph nodes is strongly recommended for any histological type of EGJ cancer, regardless of the presence or absence of neoadjuvant chemotherapy and the extent of gastric involvement. In contrast, no survival benefit was observed for the dissection of left greater curvature, distal perigastric, or paraaortic lymph nodes. Middle and lower mediastinal node

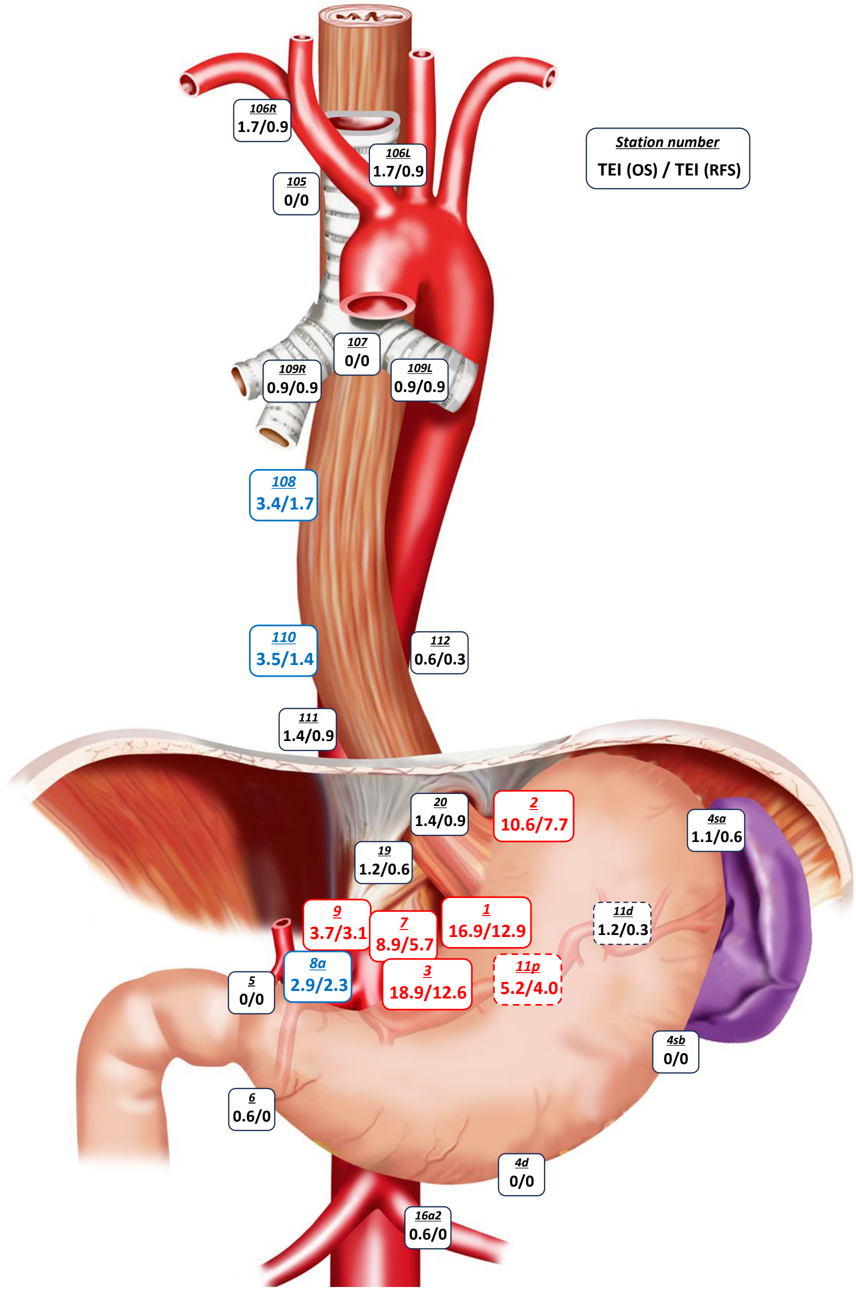

Figure 3. The therapeutic efficacy indices (TEIs) for 5-year OS and recurrence-free survival (RFS) in mediastinal and abdominal nodes

Stations in red indicate indices >3 for 5-year OS and >2 for 5-year RFS, while stations in blue indicate indices >3 for 5-year OS or >2 for 5-year RFS.

dissection should be considered only when esophageal involvement exceeded 3 and 2 cm, respectively.

Our initial analysis in this prospective study identified the distribution of lymph node metastases in EGJ cancer.[14] Since most prior studies were retrospective, they could not accurately determine the metastasis rate of each lymph node. Indeed, the metastasis rates in mediastinal and paraaortic nodes observed in this prospective study were lower than those reported in previous retrospective studies.[26–28] This discrepancy likely stems from selection bias inherent in routine clinical practice, wherein surgeons tend to dissect lymph nodes they suspect to be metastatic while omitting those they believe to be nonmetastatic. In our initial report, we proposed a provisional algorithm for lymph node dissection and the surgical approach to EGJ cancer based on the metastasis rate of each lymph node. However, a critical limitation of that algorithm was that it did not account for the actual impact of lymph node dissection on survival. In other words, even if a lymph node had a high metastasis rate, dissection would have limited significance if recurrence occurred soon afterward. Therefore, the lymph nodes that should be dissected are those where dissection is more likely to contribute to long-term survival. Ideally, an RCT would be conducted in which all patients undergo a subtotal esophagectomy via the RT approach, with a control group receiving standard lymphadenectomy and an experimental group receiving either extended or limited lymphadenectomy. However, two major issues made such an RCT infeasible. First, as established by the JCOG9502 trial conducted in Japan in the 1990s, lower esophagectomy via the abdominal TH approach is the standard for EGJ adenocarcinomas with $\leq$3 cm of esophageal involvement, and this is clearly stated in the Japanese Gastric Cancer Treatment Guidelines.[17] Therefore, performing a subtotal esophagectomy via the RT approach in such cases is generally not acceptable in Japan unless the patient is diagnosed preoperatively with cN+ in the upper or middle mediastinum. Second, even when performing a subtotal esophagectomy via the RT approach, there is no consensus on the standard extent of mediastinal lymphadenectomy, with substantial variation among institutions and even within the same institution depending on whether the surgeon is a gastric or thoracic specialist. As a second-best alternative for determining the optimal extent of dissection, we conducted a prospective analysis using TEI proposed by Sasako et al.,[19] which has been used to establish standard lymphadenectomy in various areas in recent years.[21–25] Indeed, the use of the TEI cannot yield conclusions at the same level of evidence as an RCT. However, using the TEI determined in this study for EGJ cancer, we have been able to propose a provisional standard surgical procedure. We

Table 2. Therapeutic efficacy indices for 5-year overall survival and recurrence-free survival in the mediastinal nodes according to the length of esophageal involvement

	Esophageal involvement[b]	
	≤3.0 cm	>3.0 cm
Lymph node station	OS/RFS	OS/RFS
Upper mediastinal nodes[a]	(*n* = 72)	(*n* = 45)
105	0/0	0/0
106L	2.8/1.4	0/0
106R	1.4/0	2.2/**2.2**
Middle mediastinal nodes[a]	(*n* = 72)	(*n* = 45)
107	0/0	0/0
108	1.4/1.4	**6.7**/**2.2**
109L	1.4/1.4	0/0
109R	0/0	2.2/**2.2**

	Esophageal involvement[b]			
	≤1.0 cm	1.1–2.0 cm	2.1–3.0 cm	>3.0 cm
	OS/RFS	OS/RFS	OS/RFS	OS/RFS
Lower mediastinal nodes	(*n* = 103)	(*n* = 103)	(*n* = 81)	(*n* = 60)
110	1.0/1.0	2.9/1.0	**4.9**/**2.5**	**6.7**/1.7
111	1.0/1.0	1.0/0	2.5/**2.5**	1.7/0
112	0/0	0/0	1.2/1.2	1.7/0

Indices in bold indicate values greater than 3 for 5-year overall survival and greater than 2 for 5-year RFS.

105, upper thoracic para-esophageal node; 106L, left recurrent laryngeal nerve node; 106R, right recurrent laryngeal nerve node; 107, subcarinal node; 108, middle thoracic para-esophageal node; 109L, left main bronchus node; 109R, right main bronchus node; 110, lower thoracic para-esophageal node; 111, supra-diaphragmatic node; 112, posterior mediastinal node.

[a]Indices in the upper or middle mediastinal nodes were evaluated in 117 patients who underwent subtotal esophagectomy via right transthoracic approach.

[b]The length of esophageal involvement was measured in the resected specimen.

hope that, in the future, an RCT will be conducted to provide higher-level evidence and ultimately establish a definitive standard surgical approach.

Our previous analysis demonstrated that the metastasis rate of mediastinal nodes varied significantly depending on the length of esophageal involvement.[14] In this analysis, we further found that the effectiveness of mediastinal node dissection was also strongly influenced by the length of esophageal involvement. We observed that in patients who received neoadjuvant chemotherapy, the post-treatment measurement of esophageal involvement length correlated more strongly with TEI than the pretreatment measurement (data not shown). This is likely because the post-treatment measurement reflects the therapeutic effects of preoperative neoadjuvant chemotherapy, making it more relevant for evaluating TEI. Consequently, the cutoff values for esophageal involvement length to perform middle mediastinal node dissection were revised from 4 to 3 cm. Furthermore, although metastases to the left greater curvature or distal perigastric nodes have been reported to occur more frequently as gastric involvement length and tumor size increase,[14,29] the TEI for these nodes remained near zero, regardless of gastric involvement length. This indicates that, even in the presence of metastases, lymphadenectomy of the left greater curvature or distal perigastric nodes provides no clear therapeutic benefit and supports the current Japanese guidelines, which state that total gastrectomy is unnecessary for tumor whose epicenter is located within 2 cm from EGJ.

Interestingly, in patients who received neoadjuvant chemotherapy, the TEI was lower for most lymph nodes than it was in those who did not. Since upper mediastinal node dissection increases the risk of recurrent laryngeal nerve palsy, a Western strategy—such as neoadjuvant chemotherapy followed by the Ivor-Lewis procedure without upper mediastinal node dissection—may be a reasonable approach even when esophageal involvement exceeds 3 cm. However, the subgroup analysis for patients diagnosed with cN+ in the upper or middle mediastinal nodes indicated the need for therapeutic (not prophylactic) dissection of upper mediastinal nodes. Additionally, our study showed that postoperative adjuvant chemotherapy increased the TEI in most nodes. This finding supports the Eastern strategy, emphasizing that sufficient adjuvant chemotherapy is desirable to improve long-term outcomes in patients with regional lymph node metastases, even after complete resection.

Based on the final analysis of this prospective study, we updated the recommended algorithm for a standardized lymph node dissection and surgical approach strategy for EGJ cancer. Proximal perigastric and suprapancreatic lymph nodes should be dissected for all types of EGJ cancer. In contrast, total gastrectomy is unnecessary as long as an adequate distal tumor margin can be preserved, and dissection of paraaortic lymph nodes is not recommended. The extent of mediastinal node dissection should be tailored to the length of esophageal involvement. To implement this strategy, an RT approach is required for tumors with esophageal involvement exceeding 3 cm, while a TH approach is sufficient for tumors with esophageal involvement of 2 cm or less. For tumors with esophageal involvement between 2 cm and 3 cm, either surgical approach is acceptable, allowing for flexibility in clinical decision-making.

Limitations of the study

This study has several limitations. First, although this is the largest prospective study evaluating surgical strategies for EGJ cancer, the number of patients who underwent thorough mediastinal node dissection was relatively small. Therefore, caution is needed when interpreting the TEI for upper or middle mediastinal nodes, and subgroup analysis findings should also be interpreted as exploratory. Second, this study included both histological types—adenocarcinoma and SCC—which may differ in their biological behavior. However, recent landmark trials in perioperative treatment for EGJ cancer (CROSS, NeoRes, CheckMate-577) have considered these two histological types within the same therapeutic framework.[11–13] Furthermore, our findings that the long-term survival and TEI did not substantially differ between adenocarcinoma and SCC support this integrated approach. Third, no standard TEI threshold exists to determine the indication for lymph node dissection. In the original study by Sasako et al., the TEI for 5-year OS in most nodes dissected

Table 3. Therapeutic efficacy indices for 5-year overall survival and recurrence-free survival in the mediastinal and abdominal nodes by histological type, neoadjuvant, and adjuvant treatment status

	Histology		Neoadjuvant treatment		Adjuvant treatment	
	AD	SCC	No	Yes	No	Yes
Lymph node station	OS/RFS	OS/RFS	OS/RFS	OS/RFS	OS/RFS	OS/RFS
Upper mediastinal nodes[a]	(*n* = 86)	(*n* = 31)	(*n* = 39)	(*n* = 78)	(*n* = 65)	(*n* = 52)
105	0/0	0/0	0/0	0/0	0/0	0/0
106L	2.3/1.2	0/0	**5.1/2.6**	0/0	0/0	**3.8**/1.9
106R	2.3/1.2	0/0	2.6/**2.6**	1.3/0	1.5/0	1.9/1.9
Middle mediastinal nodes[a]	(*n* = 86)	(*n* = 31)	(*n* = 39)	(*n* = 78)	(*n* = 65)	(*n* = 52)
107	0/0	0/0	0/0	0/0	0/0	0/0
108	2.3/1.2	**6.5/3.2**	0/0	**5.1/2.6**	**3.1**/1.5	**3.8**/1.9
109L	1.2/1.2	0/0	2.6/**2.6**	0/0	1.5/1.5	0/0
109R	1.2/1.2	0/0	0/0	1.3/1.3	0/0	1.9/1.9
Lower mediastinal nodes	(*n* = 316)	(*n* = 31)	(*n* = 228)	(*n* = 119)	(*n* = 158)	(*n* = 189)
110	**3.2**/1.6	**6.5**/0	**3.9/2.2**	2.5/0	1.9/0.6	**4.8/2.1**
111	1.6/1.0	0/0	2.2/1.3	0/0	2.5/1.3	0.5/0.5
112	0.6/0.3	0/0	0.4/0.4	0.8/0	0/0	1.1/0.5
Proximal perigastric nodes	(*n* = 319)	(*n* = 31)	(*n* = 230)	(*n* = 120)	(*n* = 159)	(*n* = 191)
1	**17.6/13.8**	**9.7/3.2**	**21.3/17.0**	**8.3/5.0**	**12.6/8.8**	**20.4/16.2**
2	**10.7/7.5**	**9.7/9.7**	**12.2/9.1**	**7.5/5.0**	**8.8/8.2**	**12.0/7.3**
3	**19.4/12.5**	**12.9/12.9**	**21.7/15.2**	**13.3/7.5**	**10.1/7.5**	**26.2/16.8**
7	**8.5/5.0**	**12.9/12.9**	**9.1/6.1**	**8.3/5.0**	**5.0/3.8**	**12.0/7.3**
Left greater curvature nodes[b]	(*n* = 174)	(*n* = 0)	(*n* = 141)	(*n* = 33)	(*n* = 64)	(*n* = 110)
4sa	1.1/0.6	–/–	1.4/0.7	0/0	0/0	1.8/0.9
4sb	0/0	–/–	0/0	0/0	0/0	0/0
Distal perigastric nodes[b]	(*n* = 174)	(*n* = 0)	(*n* = 141)	(*n* = 33)	(*n* = 64)	(*n* = 110)
4d	0/0	–/–	0/0	0/0	0/0	0/0
5	0/0	–/–	0/0	0/0	0/0	0/0
6	0.6/0	–/–	0/0	**3.0**/0	0/0	0.9/0
Suprapancreatic nodes	(*n* = 319)	(*n* = 31)	(*n* = 230)	(*n* = 120)	(*n* = 159)	(*n* = 191)
8a	2.5/1.9	**6.5/6.5**	**3.1/2.2**	2.5/**2.5**	1.9/1.3	**3.7/3.1**
9	**3.8/3.1**	**3.2/3.2**	**3.9/3.5**	**3.3/2.5**	1.3/1.3	**5.8/4.7**
11p	**5.7/4.4**	0/0	**6.6/4.8**	2.5/**2.5**	1.9/1.3	**7.9/6.3**
11d	1.3/0.3	0/0	1.8/0.4	0/0	0.6/0	1.6/0.5
Abdominal hiatal nodes	(*n* = 315)	(*n* = 31)	(*n* = 226)	(*n* = 120)	(*n* = 158)	(*n* = 188)
19	1.3/0.6	0/0	1.3/0.9	0.8/0	0.6/0.6	1.6/0.5
20	1.3/1.0	**3.2**/0	1.8/1.3	0.8/0	1.3/0.6	1.6/1.1
Paraaortic nodes	(*n* = 309)	(*n* = 31)	(*n* = 222)	(*n* = 118)	(*n* = 154)	(*n* = 186)
16a2	0.6/0	0/0	0.5/0	0.8/0	0.6/0	0.5/0

AD, adenocarcinoma; SCC, squamous cell carcinoma.
Indices in bold indicate values greater than 3 for 5-year overall survival and greater than 2 for 5-year RFS.
1 indicates right para-cardial node; 2, left para-cardial node; 3, lesser curvature node; 4sa, left greater curvature node along the short gastric arteries; 4sb, left greater curvature node along the left gastroepiploic artery; 4d, right greater curvature node; 5, supra-pyloric node; 6, infra-pyloric node; 7, node along the trunk of left gastric artery; 8a, antero-superior node along the common hepatic artery; 9, celiac artery node; 11p, proximal splenic artery node; 11d, distal splenic artery node; 19, infra-diaphragmatic node predominantly along the subphrenic artery; 20, para-esophageal node in the diaphragmatic esophageal hiatus; 16a2, paraaortic node between the upper margin of the origin of the celiac artery and the lower border of the left renal vein; 105, upper thoracic para-esophageal node; 106L, left recurrent laryngeal nerve node; 106R, right recurrent laryngeal nerve node; 107, subcarinal node; 108, middle thoracic para-esophageal node; 109L, left main bronchus node; 109R, right main bronchus node; 110, lower thoracic para-esophageal node; 111, supra-diaphragmatic node; 112, posterior mediastinal node.
[a]Indices in the upper or middle mediastinal nodes were evaluated in 117 patients who underwent subtotal esophagectomy via right transthoracic approach.
[b]Indices in the left greater curvature or distal perigastric nodes were evaluated in 174 patients who underwent total gastrectomy.

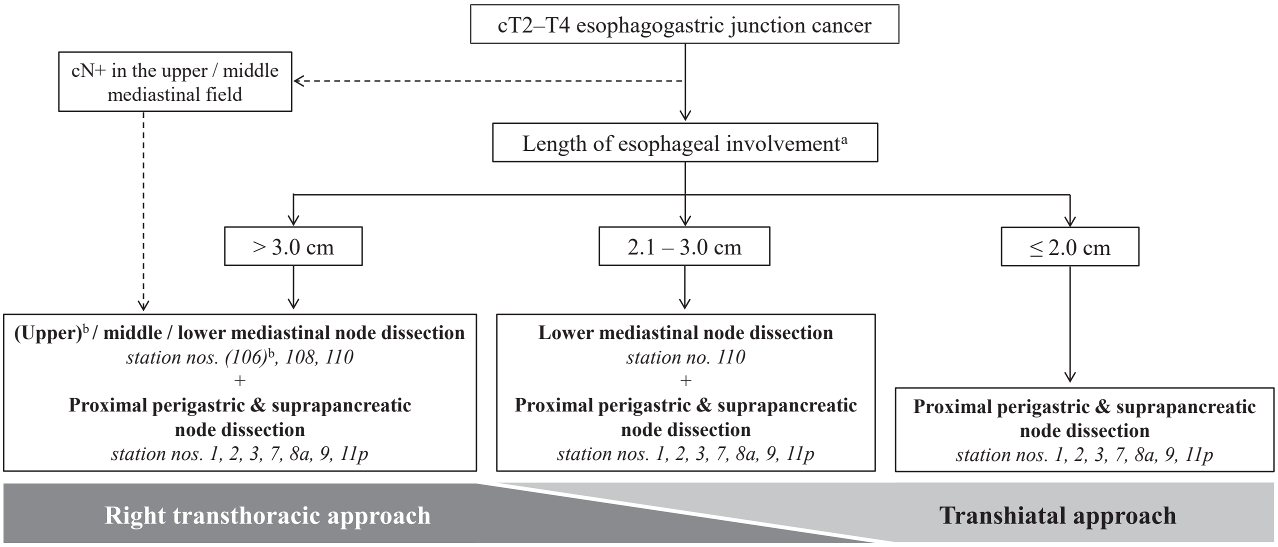

Figure 4. Recommended algorithm for the extent of lymph node dissection and the surgical approach to cT2–T4 esophagogastric junction cancer

[a]If preoperative chemotherapy is performed, the length of esophageal involvement should be measured after the treatment. [b]Dissection should be considered only for patients with cN+ in the upper or middle mediastinal field or for those who did not receive neoadjuvant chemotherapy.

by standard D2 lymphadenectomy for gastric cancer exceeded 1 or 2.[19,20] However, the original study used retrospective data from 1972 to 1986, whereas our study collected prospective data between 2014 and 2017. Recent advances in chemotherapy and immunotherapy have enabled many cancer-bearing patients to survive beyond 5 years, with 18.9% of our cohort surviving beyond 5 years after recurrence. For this reason, we believe the TEI should be evaluated for both 5-year OS and 5-year RFS. Naturally, the appropriate threshold should balance the risks and benefits of dissection for each cancer type. Considering the risks associated with regional lymph node dissection in EGJ cancer, we reached a consensus to set the TEI threshold at 3 for 5-year OS and at 2 for 5-year RFS, meaning that a $\geq$3% improvement in 5-year OS or $\geq$2% improvement in 5-year RFS was considered to justify dissection. Fourth, there is a potential structural bias related to the surgical approach. Because the surgical approach largely determines which lymph node stations are dissected, the detection of nodal metastasis is not independent of the extent of dissection. The study design was based on the results of the JCOG9502 trial, which demonstrated that RT esophagectomy is neither ethically nor practically acceptable for tumors with $\leq$3 cm of esophageal involvement unless upper or middle mediastinal cN+ disease is present. Accordingly, our study design inevitably reflected this clinical constraint. Last, following our recommended algorithm requires accurate preoperative assessment of esophageal involvement length. However, conventional imaging modalities such as endoscopy may not be reliable, particularly in cases with large tumors. A potential alternative is positron emission tomography-computed tomography, as our preliminary study demonstrated a high sensitivity of 97% for detecting the primary tumor of EGJ adenocarcinoma.[30] By constructing an oblique slice that includes the proximal edge of FDG uptake and the EGJ or estimating the craniocaudal distance from the proximal edge to the vena cava foramen,[31] esophageal involvement length can be measured with high accuracy.

RESOURCE AVAILABILITY

Lead contact

Requests for further information and resources should be directed to and will be fulfilled by the lead contact, Yukinori Kurokawa (ykurokawa@gesurg.med.osaka-u.ac.jp).

Materials availability

This study did not generate new unique reagents.

Data and code availability

- All data reported in this paper and any additional information required to reanalyze the data will be shared by the lead contact upon reasonable request. Specifically, de-identified individual patient-level data, including baseline clinical variables and survival outcomes, will be available upon request. Any additional information regarding individual participants that may result in a breach of patient confidentiality will not be provided.
- All analyses were performed using standard, commercially available, or open-source software, which are listed in the key resources table and described in the STAR Methods. No custom computer code was developed for this study.
- Any additional information required to reanalyze the data reported in this work paper is available from the lead contact upon request.

ACKNOWLEDGMENTS

This study was supported by the JGCA and the JES. The authors thank all the participants, their families, and the OGSG data center for data management.

CellPress
OPEN ACCESS

AUTHOR CONTRIBUTIONS

Y. Kurokawa and H.T. had the original idea for the study, and Y.D. and Y. Kitagawa chaired the study group. Y. Kurokawa wrote the protocol, assisted by H.T. and T. Shimokawa. Statistical analyses were performed by T. Shimokawa and Y. Kurokawa. All authors except T. Shimokawa recruited patients into the study. Y. Kurokawa drafted the paper. H.T., Y.D., T. Shimokawa, and Y. Kitagawa revised the paper. S.M., M.T., T. Yasuda, K.Y., H.D., S.S., T. Yoshikawa, C.K., Y.S., S.T., and T. Sano reviewed the paper. All authors approved the final version.

DECLARATION OF INTERESTS

The authors declare no competing interests.

DECLARATION OF GENERATIVE AI AND AI-ASSISTED TECHNOLOGIES IN THE WRITING PROCESS

During the preparation of this work, the authors used ChatGPT to assist in checking grammar and to generate illustrative figures in the graphical abstract. After using this tool/service, the authors reviewed and edited the content as needed and take full responsibility for the content of the publication.

STAR★METHODS

Detailed methods are provided in the online version of this paper and include the following:

- KEY RESOURCES TABLE
- EXPERIMENTAL MODEL AND STUDY PARTICIPANT DETAILS
  - Human participants
- METHOD DETAILS
  - Intervention
  - Outcomes
- QUANTIFICATION AND STATISTICAL ANALYSIS
  - Statistical analysis

SUPPLEMENTAL INFORMATION

Supplemental information can be found online at https://doi.org/10.1016/j.xcrm.2026.102627.

Received: June 8, 2025
Revised: August 18, 2025
Accepted: January 16, 2026
Published: February 17, 2026

REFERENCES

1. Mariette, C., Piessen, G., Briez, N., Gronnier, C., and Triboulet, J.P. (2011). Oesophagogastric junction adenocarcinoma: which therapeutic approach? Lancet Oncol. *12*, 296–305.
2. Kusano, C., Gotoda, T., Khor, C.J., Katai, H., Kato, H., Taniguchi, H., and Shimoda, T. (2008). Changing trends in the proportion of adenocarcinoma of the esophagogastric junction in a large tertiary referral center in Japan. J. Gastroenterol. Hepatol. *23*, 1662–1665.
3. Yamashita, H., Seto, Y., Sano, T., Makuuchi, H., Ando, N., and Sasako, M.; Japanese Gastric Cancer Association and the Japan Esophageal Society (2017). Results of a nation-wide retrospective study of lymphadenectomy for esophagogastric junction carcinoma. Gastric Cancer *20*, 69–83.
4. Yanagimoto, Y., Kurokawa, Y., and Doki, Y. (2024). Surgical and Perioperative Treatments for Esophagogastric Junction Cancer. Ann. Thorac. Cardiovasc. Surg. *30*, 24-00056–00056.
5. Yanagimoto, Y., Kurokawa, Y., and Doki, Y. (2023). Essential updates 2021/2022: Perioperative and surgical treatments for gastric and esophagogastric junction cancer. Ann. Gastroenterol. Surg. *7*, 698–708.
6. Kitagawa, Y., Matsuda, S., Gotoda, T., Kato, K., Wijnhoven, B., Lordick, F., Bhandari, P., Kawakubo, H., Kodera, Y., Terashima, M., et al. (2024). Clinical practice guidelines for esophagogastric junction cancer: Upper GI Oncology Summit 2023. Gastric Cancer *27*, 401–425.
7. Hulscher, J.B.F., van Sandick, J.W., de Boer, A.G.E.M., Wijnhoven, B.P.L., Tijssen, J.G.P., Fockens, P., Stalmeier, P.F.M., ten Kate, F.J.W., van Dekken, H., Obertop, H., et al. (2002). Extended transthoracic resection compared with limited transhiatal resection for adenocarcinoma of the esophagus. N. Engl. J. Med. *347*, 1662–1669.
8. Omloo, J.M.T., Lagarde, S.M., Hulscher, J.B.F., Reitsma, J.B., Fockens, P., van Dekken, H., Ten Kate, F.J.W., Obertop, H., Tilanus, H.W., and van Lanschot, J.J.B. (2007). Extended transthoracic resection compared with limited transhiatal resection for adenocarcinoma of the mid/distal esophagus: five-year survival of a randomized clinical trial. Ann. Surg. *246*, 992–1001.
9. Sasako, M., Sano, T., Yamamoto, S., Sairenji, M., Arai, K., Kinoshita, T., Nashimoto, A., and Hiratsuka, M.; Japan Clinical Oncology Group JCOG9502 (2006). Left thoracoabdominal approach versus abdominal-transhiatal approach for gastric cancer of the cardia or subcardia: a randomised controlled trial. Lancet Oncol. *7*, 644–651.
10. Kurokawa, Y., Sasako, M., Sano, T., Yoshikawa, T., Iwasaki, Y., Nashimoto, A., Ito, S., Kurita, A., Mizusawa, J., and Nakamura, K.; Japan Clinical Oncology Group JCOG9502 (2015). Ten-year follow-up results of a randomized clinical trial comparing left thoracoabdominal and abdominal transhiatal approaches to total gastrectomy for adenocarcinoma of the oesophagogastric junction or gastric cardia. Br. J. Surg. *102*, 341–348.
11. Shapiro, J., van Lanschot, J.J.B., Hulshof, M.C.C.M., van Hagen, P., van Berge Henegouwen, M.I., Wijnhoven, B.P.L., van Laarhoven, H.W.M., Nieuwenhuijzen, G.A.P., Hospers, G.A.P., Bonenkamp, J.J., et al. (2015). Neoadjuvant chemoradiotherapy plus surgery versus surgery alone for oesophageal or junctional cancer (CROSS): long-term results of a randomised controlled trial. Lancet Oncol. *16*, 1090–1098.
12. Klevebro, F., Alexandersson von Döbeln, G., Wang, N., Johnsen, G., Jacobsen, A.B., Friesland, S., Hatlevoll, I., Glenjen, N.I., Lind, P., Tsai, J.A., et al. (2016). A randomized clinical trial of neoadjuvant chemotherapy versus neoadjuvant chemoradiotherapy for cancer of the oesophagus or gastro-oesophageal junction. Ann. Oncol. *27*, 660–667.
13. Kelly, R.J., Ajani, J.A., Kuzdzal, J., Zander, T., Van Cutsem, E., Piessen, G., Mendez, G., Feliciano, J., Motoyama, S., Lièvre, A., et al. (2021). Adjuvant Nivolumab in Resected Esophageal or Gastroesophageal Junction Cancer. N. Engl. J. Med. *384*, 1191–1203.
14. Kurokawa, Y., Takeuchi, H., Doki, Y., Mine, S., Terashima, M., Yasuda, T., Yoshida, K., Daiko, H., Sakuramoto, S., Yoshikawa, T., et al. (2021). Mapping of Lymph Node Metastasis From Esophagogastric Junction Tumors: A Prospective Nationwide Multicenter Study. Ann. Surg. *274*, 120–127.
15. Mine, S., Kurokawa, Y., Takeuchi, H., Terashima, M., Yasuda, T., Yoshida, K., Yabusaki, H., Shirakawa, Y., Fujitani, K., Sano, T., et al. (2022). Postoperative complications after a transthoracic esophagectomy or a transhiatal gastrectomy in patients with esophagogastric junctional cancers: a prospective nationwide multicenter study. Gastric Cancer *25*, 430–437.
16. Motoori, M., Kurokawa, Y., Takeuchi, H., Sano, T., Terashima, M., Ito, S., Komatsu, S., Hosoya, Y., Hirao, M., Yamashita, K., et al. (2022). Risk Factors for Para-Aortic Lymph Node Metastasis in Esophagogastric Junction Cancer: Results from a Prospective Nationwide Multicenter Study. Ann. Surg Oncol. *29*, 5649–5654.
17. Japanese Gastric Cancer Association (2023). Japanese Gastric Cancer Treatment Guidelines 2021 (6th edition). Gastric Cancer *26*, 1–25.
18. Kitagawa, Y., Ishihara, R., Ishikawa, H., Ito, Y., Oyama, T., Oyama, T., Kato, K., Kato, H., Kawakubo, H., Kawachi, H., et al. (2023). Esophageal cancer practice guidelines 2022 edited by the Japan Esophageal Society: part 2. Esophagus *20*, 373–389.
19. Sasako, M., McCulloch, P., Kinoshita, T., and Maruyama, K. (1995). New method to evaluate the therapeutic value of lymph node dissection for gastric cancer. Br. J. Surg. *82*, 346–351.

20. Japanese Gastric Cancer Association (1998). Japanese classification of gastric carcinoma: 2nd English edition. Gastric Cancer *1*, 10–24.
21. Imamura, T., Yamamoto, Y., Sugiura, T., Okamura, Y., Ito, T., Ashida, R., Ohgi, K., and Uesaka, K. (2021). Reconsidering the Optimal Regional Lymph Node Station According to Tumor Location for Pancreatic Cancer. Ann. Surg Oncol. *28*, 1602–1611.
22. Abughararah, T.Z., Jeong, Y.H., Alabbood, F., Chong, Y., Yun, J.K., Lee, G.D., Choi, S., Kim, H.R., Kim, Y.H., Kim, D.K., and Park, S.I. (2021). Lobe-specific lymph node dissection in stage IA non-small-cell lung cancer: a retrospective cohort study. Eur. J. Cardio. Thorac. Surg. *59*, 783–790.
23. Oshikiri, T., Goto, H., Kato, T., Hasegawa, H., Kanaji, S., Yamashita, K., Fujino, Y., Tominaga, M., Matsuda, T., and Kakeji, Y. (2023). Improvement Effect of Upper Mediastinal Lymphadenectomy During Minimally Invasive Esophagectomy on the Prognosis in Squamous Cell Carcinoma: Efficacy Index and Propensity Score Matching Analyses. J. Am. Coll. Surg. *237*, 762–770.
24. Miura, Y., Ohgi, K., Ashida, R., Yamada, M., Otsuka, S., Sasaki, K., Uesaka, K., and Sugiura, T. (2024). Efficacy of lymph node dissection for duodenal cancer according to the lymph node station. Ann. Gastroenterol. Surg. *8*, 51–59.
25. Terasaki, F., Sugiura, T., Okamura, Y., Ashida, R., Ohgi, K., Yamada, M., Ohtsuka, S., and Uesaka, K. (2024). Benefit of lymph node dissection for perihilar and distal cholangiocarcinoma according to lymph node stations. J. Hepatobiliary. Pancreat. Sci. *31*, 251–261.
26. Pedrazzani, C., de Manzoni, G., Marrelli, D., Giacopuzzi, S., Corso, G., Minicozzi, A.M., Rampone, B., and Roviello, F. (2007). Lymph node involvement in advanced gastroesophageal junction adenocarcinoma. J. Thorac. Cardiovasc. Surg. *134*, 378–385.
27. Kurokawa, Y., Hiki, N., Yoshikawa, T., Kishi, K., Ito, Y., Ohi, M., Wada, N., Takiguchi, S., Mine, S., Hasegawa, S., et al. (2015). Mediastinal lymph node metastasis and recurrence in adenocarcinoma of the esophagogastric junction. Surgery *157*, 551–555.
28. Yoshikawa, T., Takeuchi, H., Hasegawa, S., Nozaki, I., Kishi, K., Ito, S., Ohi, M., Mine, S., Hara, J., Matsuda, T., et al. (2016). Theoretical therapeutic impact of lymph node dissection on adenocarcinoma and squamous cell carcinoma of the esophagogastric junction. Gastric Cancer *19*, 143–149.
29. Mine, S., Kurokawa, Y., Takeuchi, H., Kishi, K., Ito, Y., Ohi, M., Matsuda, T., Hamakawa, T., Hasegawa, S., Yoshikawa, T., and Hiki, N. (2015). Distribution of involved abdominal lymph nodes is correlated with the distance from the esophagogastric junction to the distal end of the tumor in Siewert type II tumors. Eur. J. Surg. Oncol. *41*, 1348–1353.
30. Wada, N., Kurokawa, Y., Takahashi, T., Saito, T., Yamamoto, K., Yamashita, K., Tanaka, K., Makino, T., Nakajima, K., Tatsumi, M., et al. (2023). Accurate Preoperative Evaluation of the Location of Esophagogastric Junction Adenocarcinoma Using Positron Emission Tomography-Computed Tomography. Am. Surg. *89*, 6005–6012.
31. Nagano S., Kurokawa Y., Hagi T., Saito T., Takahashi T., Nakai S., Momose K., Yamashita K., Tanaka K., Makino T., Nakajima K., Eguchi H., Doki Y. Estimation of esophageal involvement length in esophagogastric junction tumors using PET-CT. Esophagus. doi: 10.1007/s10388-026-01183-6. Online ahead of print.
32. Japanese Gastric Cancer Association (2011). Japanese classification of gastric carcinoma: 3rd English edition. Gastric Cancer *14*, 101–112.
33. Japan Esophageal Society (2017). Japanese Classification of Esophageal Cancer, 11th Edition: part I. Esophagus *14*, 1–36. 14.

CellPress
OPEN ACCESS

STAR★METHODS

KEY RESOURCES TABLE

REAGENT or RESOURCE	SOURCE	IDENTIFIER
Software and algorithms		
R version 4.2.1	R Project for Statistical Computing	https://www.r-project.org/
SPSS Statistics version 24	IBM Open Source	https://www.ibm.com/
Other		
Trial registration number: UMIN000013205	UMIN Clinical Trials Registry	https://center6.umin.ac.jp/cgi-open-bin/ctr_e/ctr_view.cgi?recptno=R000015407

EXPERIMENTAL MODEL AND STUDY PARTICIPANT DETAILS

Human participants

This was a nationwide, multicenter, prospective study conducted at 42 institutions affiliated with the JGCA and the JES. The eligibility criteria were as follows: (1) tumor epicenter located within 2 cm of the EGJ; (2) histologically confirmed adenocarcinoma, squamous cell carcinoma (SCC), or adenosquamous carcinoma; (3) clinical stage cT2–T4; (4) tumor deemed resectable; (5) patient age 20 years or older; (6) Eastern Cooperative Oncology Group (ECOG) performance status of 0–2; (7) no prior history of gastrectomy; and (8) adequate organ function. Tumor, node, and metastasis (TNM) status was classified according to the Japanese Classification of Gastric Carcinoma, 14th edition.[32] To ensure the eligibility of enrolled patients, photographs of resected specimens from all cases were centrally reviewed in regular semi-annual meetings (Figure S4). The study protocol was approved by the institutional review boards of all participating hospitals, and all patients provided written informed consent before enrollment.

METHOD DETAILS

Intervention

After confirming eligibility, patients were enrolled before surgery. Within 2 weeks after enrollment, surgeons performed either subtotal esophagectomy with upper gastrectomy via the right transthoracic (RT) approach or lower esophagectomy with total or proximal gastrectomy via the abdominal transhiatal (TH) approach, according to the study protocol. Briefly, the RT approach was selected for patients who had either adenocarcinoma with esophageal involvement over 3 cm or SCC. Otherwise, the TH approach was preferred, though the left transthoracic approach was also permitted. If a patient was clinically diagnosed with lymph node metastasis (cN+), defined as lymph nodes with a short-axis diameter $\geq$8 mm on CT or showing significant FDG uptake on PET-CT, in the upper or middle mediastinal field, the RT approach was selected to enable therapeutic dissection of these nodes regardless of histological type or the length of esophageal involvement.

All surgical approaches involved lymph node dissection in the perigastric field (station nos. 1, 2, 3a, 7), suprapancreatic field (station nos. 8a, 9, 11p, 11d), paraaortic field (station no. 16a2lat), abdominal hiatal field (station nos. 19, 20), and lower mediastinal field (station nos. 110, 111, 112). The RT approach additionally required extensive thorough mediastinal node dissection, including upper mediastinal (station nos. 105, 106L, 106R) and middle mediastinal (station nos. 107, 108, 109L, 109R) nodes. The reconstruction method was not specified in this study.

The study protocol did not mandate specific indications for neoadjuvant or adjuvant therapy, because the Japanese guidelines by the JGCA and JES explicitly state that there is no clear recommendation for perioperative treatment in resectable EGJ cancer, due to insufficient evidence in Asian populations. Video-assisted thoracic surgery, laparoscopic surgery, and hand-assisted laparoscopic surgery were permitted. Although no specific surgeon qualifications were mandated in this trial, all participating surgeons agreed upon the surgical techniques during the study planning phase to ensure technical consistency. Additionally, regular monitoring reports were reviewed, and surgical videos were analyzed in meetings to standardize operative procedures. Lymph node stations were classified according to the Japanese Classification of Gastric Carcinoma, 14th edition and the Japanese Classification of Esophageal Cancer, 11th edition.[32,33]

Outcomes

The primary endpoint was the metastasis rate at each lymph node station in all eligible patients. Secondary endpoints included the R0 resection rate, postoperative complications, overall survival (OS), recurrence-free survival (RFS), recurrence sites, and the TEI. The TEI was calculated by multiplying the lymph node metastasis rate at each station by the 5-year OS or RFS rate among patients with

metastases. The metastasis rate for each station was calculated as the number of eligible patients with pathological metastases in that station divided by the number of eligible patients who underwent dissection of that station. OS was defined as the time from surgery to death from any cause, while RFS was defined as the time from surgery to the first recurrence or death from any cause. Patients lost to follow-up within 5 years were excluded from the TEI calculation.

QUANTIFICATION AND STATISTICAL ANALYSIS

Statistical analysis

Based on an assumed 15% metastasis rate in the upper or middle mediastinal nodes and a 95% CI width of ≤15%, the estimated sample size required for the RT approach was 100 patients. Since approximately twice as many patients were expected to be eligible for the TH approach, the total required sample size was 300. Considering a potential 15% rate of ineligible or unevaluable cases, the final target sample size was set at 360 patients (120 for the RT approach and 240 for the TH approach).

Metastasis rates and their 95% CIs were estimated using the Clopper–Pearson exact method. OS and RFS curves were estimated using the Kaplan–Meier method and compared using an unstratified log rank test. All statistical analyses were conducted using R version 4.2.1 (R Foundation for Statistical Computing, Vienna, Austria) or SPSS Statistics version 24 (IBM, Armonk, NY, USA).

Cell Reports Medicine, Volume *7*

Supplemental information

Establishing a standard surgery

for esophagogastric junction cancer: Final results

from the JGCA-JES nationwide prospective study

Yukinori Kurokawa, Hiroya Takeuchi, Yuichiro Doki, Shinji Mine, Masanori Terashima, Takushi Yasuda, Kazuhiro Yoshida, Hiroyuki Daiko, Shinichi Sakuramoto, Takaki Yoshikawa, Chikara Kunisaki, Yasuyuki Seto, Shigeyuki Tamura, Toshio Shimokawa, Takeshi Sano, and Yuko Kitagawa

Supplemental information

Establishing a standard surgery for esophagogastric junction cancer: Final results from the JGCA-JES nationwide prospective study

Yukinori Kurokawa, Hiroya Takeuchi, Yuichiro Doki, Shinji Mine, Masanori Terashima, Takushi Yasuda, Kazuhiro Yoshida, Hiroyuki Daiko, Shinichi Sakuramoto, Takaki Yoshikawa, Chikara Kunisaki, Yasuyuki Seto, Shigeyuki Tamura, Toshio Shimokawa, Takeshi Sano, Yuko Kitagawa

Table S1. Site of first recurrence in 142 patients with recurrence

Sites	n (%)
Lymph nodes	63 (44.4 %)
Abdominal paraaortic	39 (27.5 %)
Mediastinal	22 (15.5 %)
Cervical	11 (7.7 %)
Perigastric	7 (4.9 %)
Others	5 (3.5 %)
Liver	37 (26.1 %)
Peritoneum	31 (21.8 %)
Lung	30 (21.1 %)
Pleura	11 (7.7 %)
Bone	9 (6.3 %)
Anastomotic site	7 (4.9 %)
Adrenal gland	5 (3.5 %)
Brain	4 (2.8 %)
Remnant esophagus	3 (2.1 %)
Others	4 (2.8 %)

Some cases are counted in multiple sites.

Table S2. Metastasis rates and therapeutic efficacy indices in the mediastinal and abdominal nodes the abdominal nodes

	Metastasis Rate (%)	5-year OS in metastatic cases (%)	TEI for OS	5-year RFS in metastatic cases (%)	TEI for RFS
Upper mediastinal nodes [a]	(n = 117)				
105	0.9	0.0	0	0.0	0
106L	2.6	66.7	1.7	33.3	0.9
106R	5.1	33.3	1.7	16.7	0.9
Middle mediastinal nodes [a]	(n = 117)				
107	2.6	0.0	0	0.0	0
108	6.8	50.0	**3.4**	25.0	1.7
109L	3.4	25.0	0.9	25.0	0.9
109R	3.4	25.0	0.9	25.0	0.9
Lower mediastinal nodes	(n = 347)				
110	9.2	37.5	**3.5**	15.6	1.4
111	3.5	41.7	1.4	25.0	0.9
112	2.0	28.6	0.6	14.3	0.3
Proximal perigastric nodes	(n = 350)				
1	34.9	48.4	**16.9**	36.9	**12.9**
2	27.1	38.9	**10.6**	28.4	**7.7**
3	37.1	50.8	**18.9**	33.8	**12.6**
7	23.7	37.3	**8.9**	24.1	**5.7**
Left greater curvature nodes [b]	(n = 174)				
4sa	4.6	25.0	1.1	12.5	0.6
4sb	1.1	0.0	0	0.0	0
Distal perigastric nodes [b]	(n = 174)				
4d	1.7	0.0	0	0.0	0
5	1.1	0.0	0	0.0	0
6	1.7	33.3	0.6	0.0	0
Suprapancreatic nodes	(n = 350)				
8a	7.2	40.0	2.9	32.0	**2.3**
9	12.3	30.2	**3.7**	25.6	**3.1**
11p	13.8	37.5	**5.2**	29.2	**4.0**
11d	4.3	26.7	1.2	6.7	0.3
Abdominal hiatal nodes	(n = 346)				
19	5.2	22.2	1.2	11.1	0.6
20	4.0	35.7	1.4	21.4	0.9

Paraaortic nodes	(n = 340)				
16a2	4.4	13.3	0.6	0.0	0

TEI, therapeutic efficacy index; OS, overall survival; RFS, recurrence-free survival.

Indices in bold indicate values greater than 3 for 5-year OS and 2 for 5-year RFS.

[a] Values in the upper or middle mediastinal nodes were evaluated in 117 patients who underwent subtotal esophagectomy via right transthoracic approach.

[b] Values in the left greater curvature or distal perigastric nodes were evaluated in 174 patients who underwent total gastrectomy.

1 indicates right para-cardial node; 2, left para-cardial node; 3, lesser curvature node; 4sa, left greater curvature node along the short gastric arteries; 4sb, left greater curvature node along the left gastroepiploic artery; 4d, right greater curvature node; 5, supra-pyloric node; 6, infra-pyloric node; 7, node along the trunk of left gastric artery; 8a, antero-superior node along the common hepatic artery; 9, celiac artery node; 11p, proximal splenic artery node; 11d, distal splenic artery node; 19, infra-diaphragmatic node predominantly along the subphrenic artery; 20, para-esophageal node in the diaphragmatic esophageal hiatus; 16a2, para-aortic node between the upper margin of the origin of the celiac artery and the lower border of the left renal vein; 105, upper thoracic para-esophageal node; 106L, left recurrent laryngeal nerve node; 106R, right recurrent laryngeal nerve node; 107, subcarinal node; 108, middle thoracic para-esophageal node; 109L, left main bronchus node; 109R, right main bronchus node; 110, lower thoracic para-esophageal node; 111, supra-diaphragmatic node; 112, posterior mediastinal node.

Table S3. Therapeutic efficacy indices for 5-year overall survival and recurrence-free survival in the abdominal nodes according to the length of gastric involvement

Lymph node station	Gastric involvement [b]		
	≤2.0 cm	2.1–4.0 cm	>4.0 cm
	OS / RFS	OS / RFS	OS / RFS
Proximal perigastric nodes	(n = 145)	(n = 141)	(n = 64)
1	**16.6** / **11.0**	**14.9** / **12.1**	**21.9** / **18.8**
2	**11.0** / **7.6**	**11.3** / **8.5**	**7.8** / **6.3**
3	**17.2** / **10.3**	**19.9** / **13.5**	**20.3** / **15.6**
7	**8.3** / **5.5**	**9.2** / **5.7**	**9.4** / **6.3**
Left greater curvature nodes [a]	(n = 44)	(n = 78)	(n = 52)
4sa	0 / 0	1.3 / 1.3	1.9 / 0
4sb	0 / 0	0 / 0	0 / 0
Distal perigastric nodes [a]	(n = 44)	(n = 78)	(n = 52)
4d	0 / 0	0 / 0	0 / 0
5	0 / 0	0 / 0	0 / 0
6	0 / 0	1.3 / 0	0 / 0
Suprapancreatic nodes	(n = 145)	(n = 141)	(n = 64)
8a	2.8 / **2.8**	**3.6** / **2.1**	1.6 / 1.6
9	2.8 / 1.4	**5.7** / **5.7**	1.6 / 1.6
11p	2.1 / **2.1**	**5.7** / **4.3**	**10.9** / **7.8**
11d	0 / 0	0.7 / 0	**4.7** / 1.6
Abdominal hiatal nodes	(n = 145)	(n = 138)	(n = 63)
19	2.1 / 0.7	0 / 0	1.6 / 1.6
20	0.7 / 0	2.9 / **2.2**	0 / 0
Paraaortic nodes	(n = 141)	(n = 137)	(n = 62)
16a2	0.7 / 0	0 / 0	1.6 / 0

OS, overall survival; RFS, recurrence-free survival.

Indices in bold indicate values greater than 3 for 5-year OS and greater than 2 for 5-year RFS.

[a] Indices in the left greater curvature or distal perigastric nodes were evaluated in 174 patients who underwent total gastrectomy.

[b] The length of gastric involvement was measured in the resected specimen.

1 indicates right para-cardial node; 2, left para-cardial node; 3, lesser curvature node; 4sa, left greater curvature node along the short gastric arteries; 4sb, left greater curvature node along the left gastroepiploic artery; 4d, right greater curvature node; 5, supra-pyloric node; 6, infra-pyloric node; 7, node along the trunk of left gastric artery; 8a, antero-superior node along the common hepatic artery; 9, celiac artery node; 11p, proximal splenic artery node; 11d, distal splenic artery node; 19, infra-diaphragmatic node predominantly along the subphrenic artery; 20, para-esophageal node in the diaphragmatic esophageal hiatus; 16a2, para-aortic node between the upper margin of the origin of the celiac artery and the lower border of the left renal vein.

Table S4. Therapeutic efficacy indices for 5-year overall survival and recurrence-free survival in patients with cN+ in the upper or middle mediastinal field

	OS / RFS
Upper mediastinal nodes	(n = 30)
105	0 / 0
106L	**6.7** / **3.3**
106R	**3.3** / 0
Middle mediastinal nodes	(n = 30)
107	0 / 0
108	**10.0** / **6.7**
109L	0 / 0
109R	**3.3** / **3.3**

OS, overall survival; RFS, recurrence-free survival.

Indices in bold indicate values greater than 3 for 5-year OS and greater than 2 for 5-year RFS.

105 indicates upper thoracic para-oesophageal node; 106L, left recurrent laryngeal nerve node; 106R, right recurrent laryngeal nerve node; 107, subcarinal node; 108, middle thoracic para-oesophageal node; 109L, left main bronchus node; 109R, right main bronchus node.

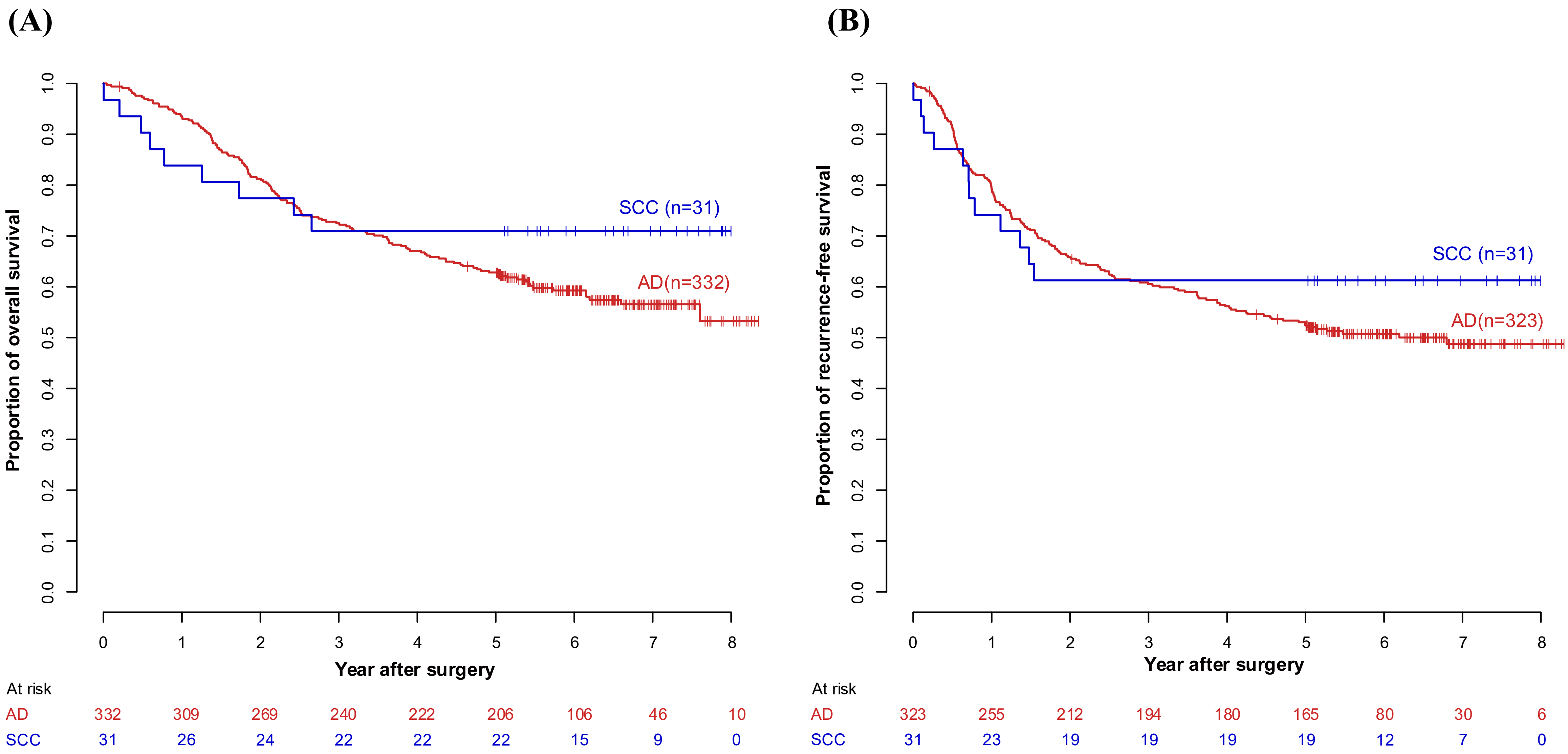

Figure S1. Overall survival in all eligible patients (A) and recurrence-free survival in all eligible patients who achieved R0 or R1 resection (B) by histological types. AD, adenocarcinoma; SCC, squamous cell carcinoma.

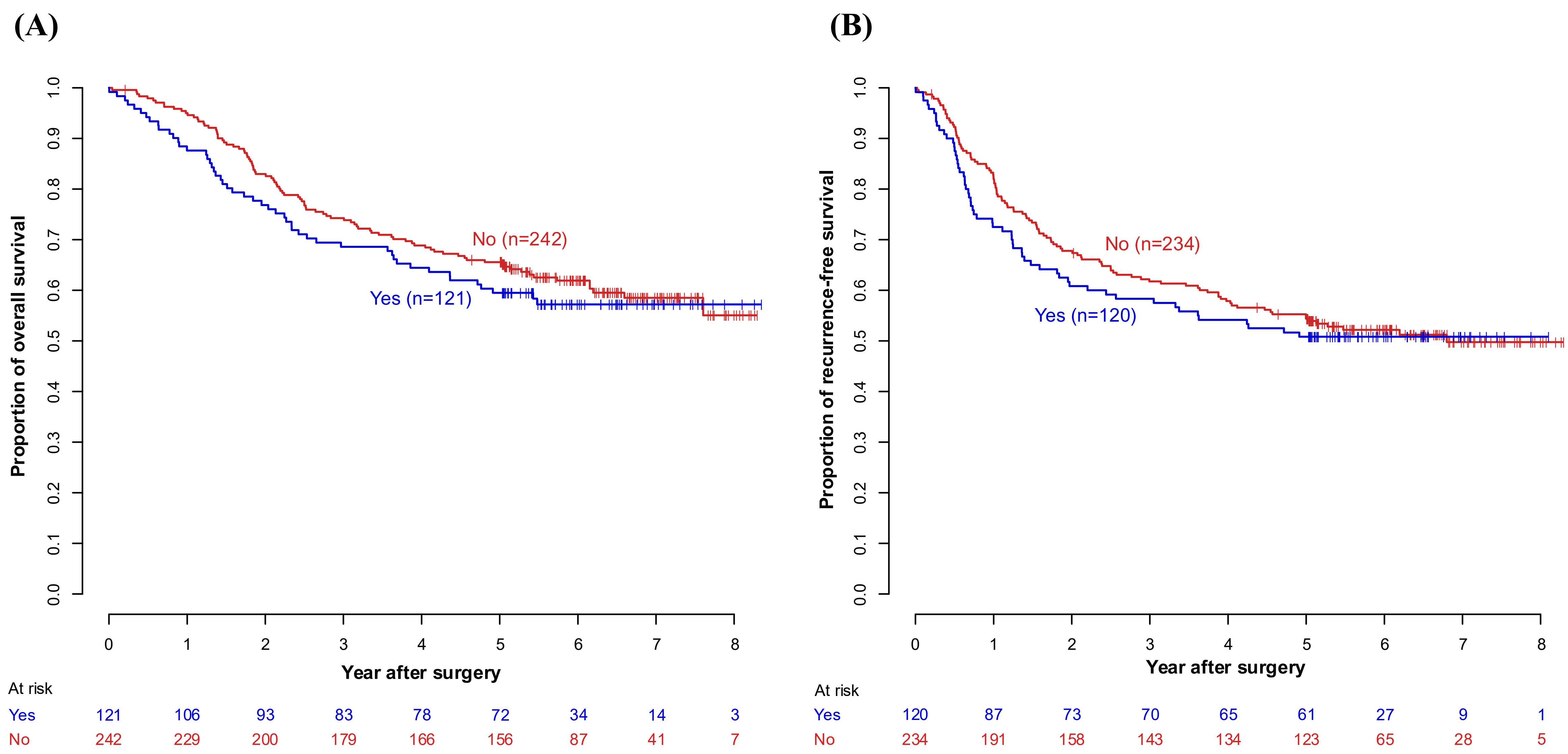

Figure S2. Overall survival in all eligible patients (A) and recurrence-free survival in all eligible patients who achieved R0 or R1 resection (B) by the presence or absence of neoadjuvant chemotherapy.

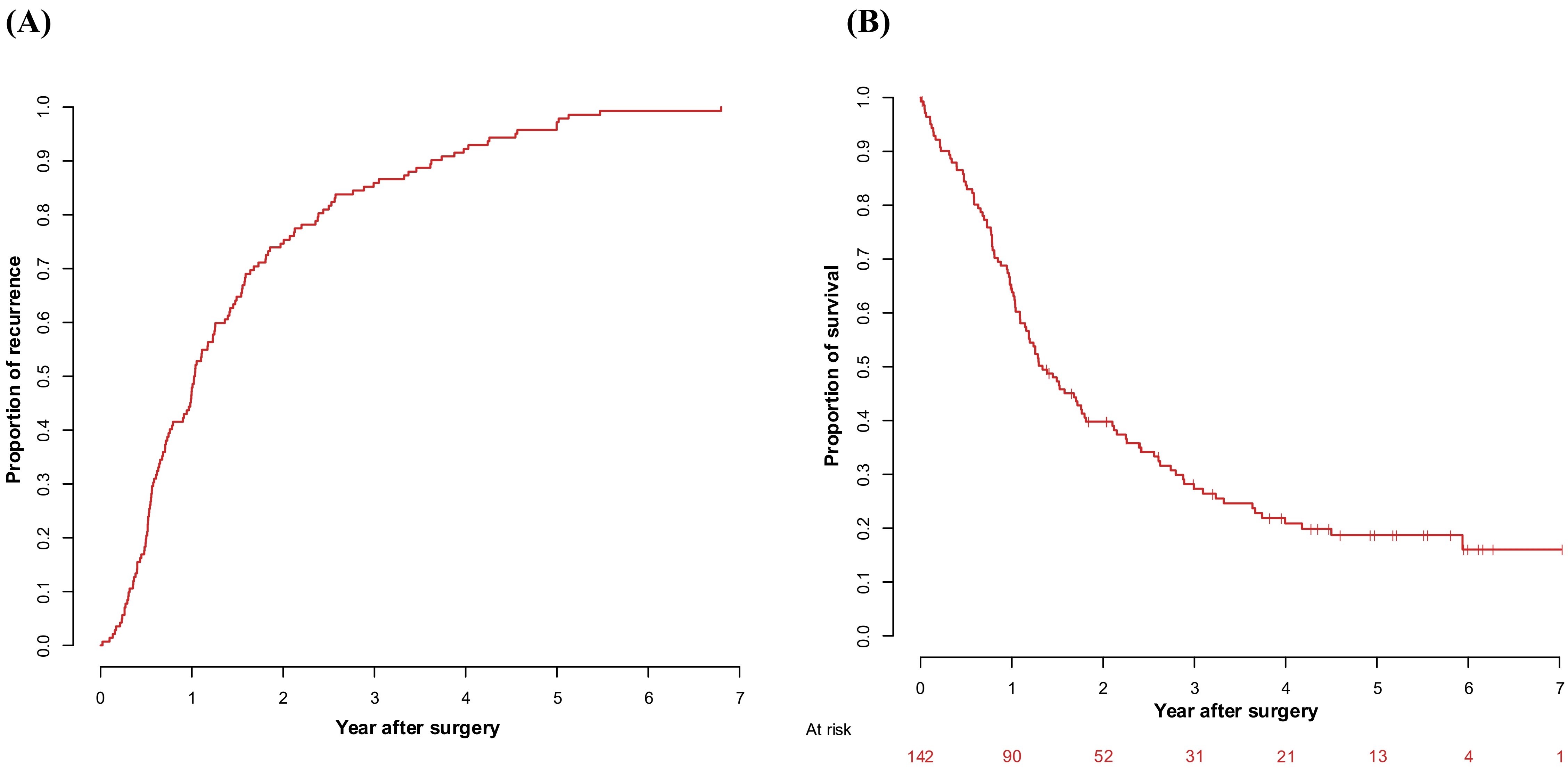

Figure S3. Cumulative recurrence rate (A) and proportion of survival after recurrence (B) in patients who experienced tumor recurrence.

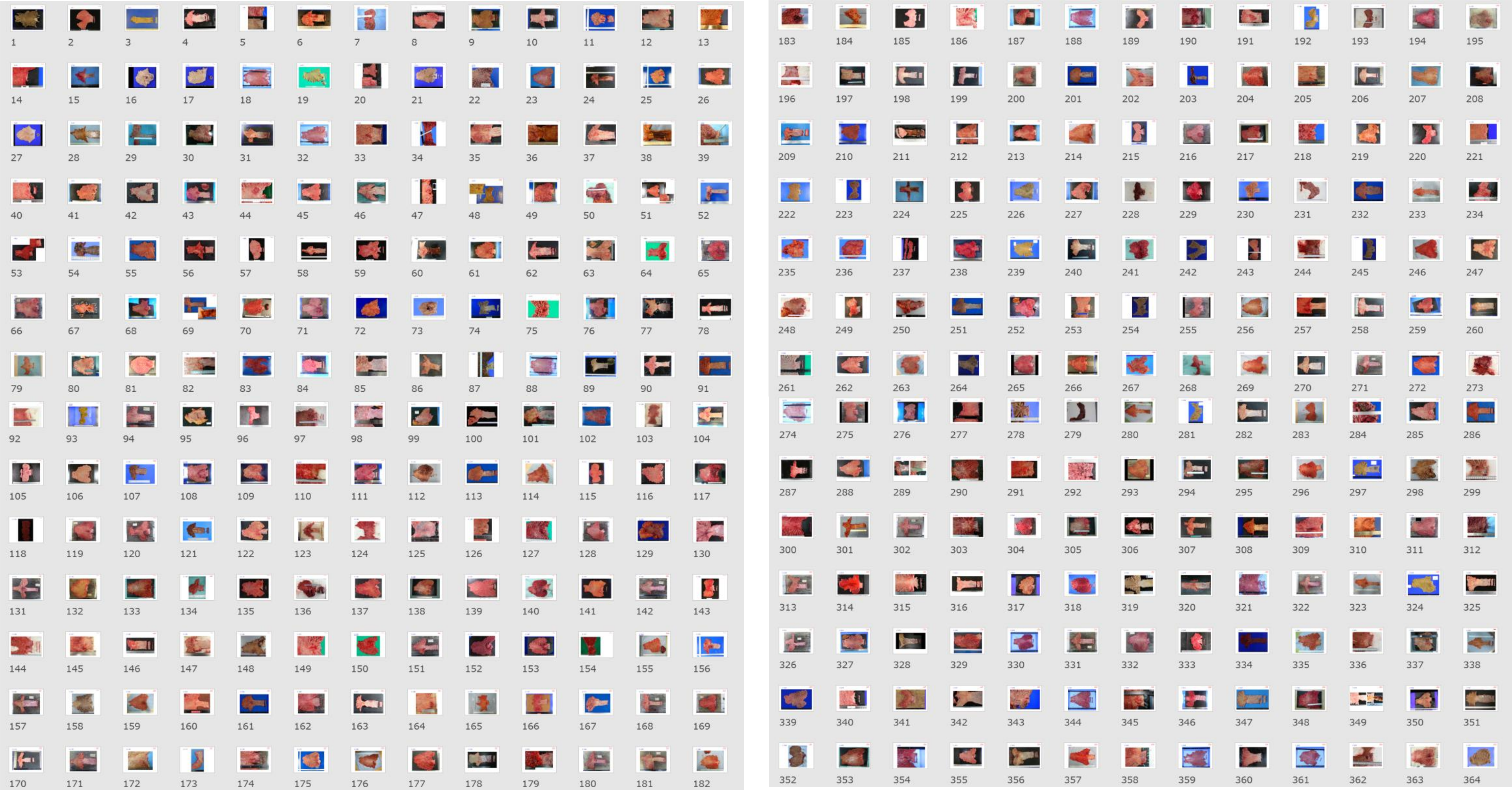

Figure S4. Representative photographs of resected specimens from each case, collected for central eligibility review.
